# Dual‐Targeted M1 Macrophage Extracellular Vesicles Reprogram the Tumor Microenvironment and Enhance Natural Killer Cell Immunotherapy in Breast Cancer

**DOI:** 10.1002/jev2.70379

**Published:** 2026-09-30

**Authors:** Su Jin Kang, Gichan Baek, Suwun Ju, Gunhee Kim, Seongjin Gwak, Won Jong Rhee

**Affiliations:** ^1^ Department of Bioengineering and Nano‐Bioengineering Incheon National University Incheon South Korea; ^2^ Division of Bioengineering Incheon National University Incheon South Korea; ^3^ Research Center for Bio Materials & Process Development Incheon National University Incheon South Korea; ^4^ Integrated Major in Bio‐manufacturing AX Incheon National University Incheon South Korea

**Keywords:** breast cancer immunotherapy, dual‐targeted, extracellular vesicles, natural killer cell, tumor microenvironment

## Abstract

Breast cancer is the most commonly diagnosed cancer worldwide and a leading cause of cancer‐related mortality in women. Despite therapeutic advances, treating advanced or recurrent cases is substantially hampered by drug resistance and the immunosuppressive tumor microenvironment (TME). Here, we report a breakthrough immunotherapeutic strategy using dual‐targeted extracellular vesicles from pro‐inflammatory M1 macrophages, hyaluronic acid (HA), and cyclic RGD (M1EV_HA/cRGD), which function as molecular bridges to physically link natural killer (NK) cells with cancer cells. Our platform simultaneously reprograms the hostile TME while activating potent antitumor immunity. HA is incorporated to engage CD44 receptors on NK cells and cRGD peptides to bind tumor‐overexpressing integrins, establishing precision dual‐targeting. M1EV_HA/cRGD could physically tether NK cells directly to tumors, and deliver inflammatory cytokines and miRNAs that transform the immunosuppressive TME into a pro‐inflammatory battlefield, substantially amplifying immune activation. *In vitro* and *in vivo* studies demonstrate that M1EV_HA/cRGD significantly enhances NK cell clustering at tumor sites, activation status, and cytotoxic killing of breast cancer cells. Unlike single‐targeted approaches, this dual‐targeting mechanism achieves simultaneous TME reprogramming and enhanced immune‐tumor engagement. M1EV_HA/cRGD is a paradigm shift in solid tumor immunotherapy that directly addresses breast cancer treatment failure, can overcome therapeutic resistance, and substantially improve patient survival outcomes.

## Graphical abstract

Dual‐targeted extracellular vesicles (EV) engineered from M1 macrophages (M1EV_HA/cRGD) bridge NK cells to breast cancer cells while activating pro‐inflammatory programs within the tumor microenvironment. Hyaluronic acid (HA) enables CD44‐mediated NK targeting, and cRGD binds tumor integrins for targeted dual engagement. These EV deliver inflammatory cytokines and miRNAs, enhancing NK cell clustering, activation, and tumor killing. *In vitro* and *in vivo*, M1EV_HA/cRGD drives potent antitumor immunity, offering a promising strategy to overcome resistance in solid tumors.

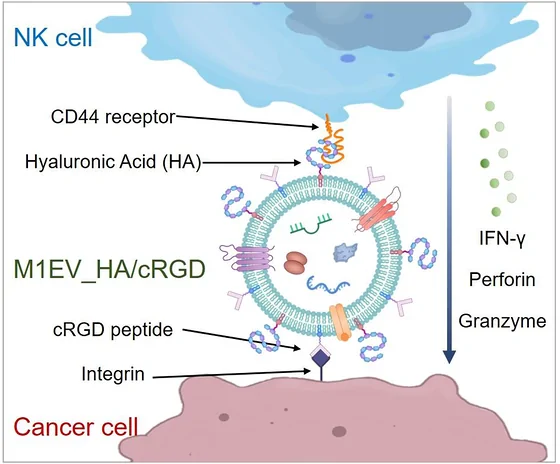

## 1 Introduction

Breast cancer is the most commonly diagnosed cancer worldwide and the leading cause of cancer‐related mortality among women (Siegel et al. 2023). Despite advances in surgery, chemotherapy, and radiotherapy, the treatment of advanced or recurrent breast cancer remains challenging due to therapeutic resistance and the immunosuppressive tumor microenvironment (TME). Traditional chemotherapy is often associated with substantial side effects that limit its use, whereas cancer immunotherapy offers targeted killing of malignant cells with fewer side effects by using immune cells (Basak et al. 2021; Juthani et al. 2024; Nurgali et al. 2018). Among the current immunotherapeutic strategies, chimeric antigen receptor T cell (CAR‐T) therapy has considerable success with hematologic malignancies but faces substantial limitations with solid tumors, including cytokine release syndrome and neurotoxicity (Sterner and Sterner 2021).

The primary obstacle in solid tumors is the TME, an intricate network of diverse cellular components, including tumor and immune cells, such as tumor‐associated macrophages (TAMs) and natural killer (NK) cells (Baghban et al. 2020; de Visser and Joyce 2023; Anderson and Simon 2020). The TME plays a critical role in cancer progression, with macrophages exhibiting complex and evolving functions. Early in tumor development, macrophages are primarily polarized to the M1 phenotype, contributing to an antitumor immune response. However, as the tumor progresses, these macrophages undergo a phenotypic shift, transforming into M2‐like TAMs, which promote angiogenesis and tumor growth (Lin et al. 2019; Au et al. 2020). Accumulation of M2 macrophages suppresses T‐cell cytotoxicity and activates regulatory T cells (T_regs_), contributing to immune resistance, which is a major hurdle in effective immunotherapy. Consequently, strategies aimed at repolarizing M2‐like TAMs to the M1 phenotype are becoming increasingly important for enhancing cancer immunotherapy. In addition, NK cells are an attractive alternative to CAR‐T cells as they do not require the recognition of human leukocyte antigens (Kim et al. 2019; Myers and Miller 2021; Laskowski et al. 2022). However, CAR‐NK cells face critical challenges with solid tumors, as NK cells have a limited capacity to penetrate the TME and selectively target specific cancers.

To overcome these limitations, we developed extracellular vesicle (EV)‐based nanoplatform using EVs derived from pro‐inflammatory M1 macrophages (M1EV) for breast cancer immunotherapy (Kim et al. 2022; Yang et al. 2024). EVs are nanoscale, membrane‐enclosed particles that mediate intercellular communication by transporting nucleic acids, proteins, lipids, and cytokines from their parent cells (Lotvall et al. 2014; Thery et al. 2018; Welsh et al. 2024; Andaloussi et al. 2013; Shekari et al. 2023). Their biocompatibility, low immunogenicity, and inherent capacity for intercellular communication make them attractive candidates for therapeutic delivery systems (Van Der Meel et al. 2019; Zhang et al. 2023b; Crescitelli et al. 2013; Jo et al. 2025; Jung et al. 2024; Park et al. 2025). M1EVs serve as immune modulators that deliver inflammatory cytokines and microRNAs (miRNAs) into the TME, thereby reprogramming macrophage polarization from the M2 to M1 phenotype. This reprogramming creates an inflammatory TME that suppresses angiogenesis and promotes tumor cell death (Tang et al. 2023; Wang et al. 2022; Ding et al. 2021; Gunassekaran et al. 2021; Yan et al. 2023; Choo et al. 2018; Zhang et al. 2023a). To address the targeting limitations of NK cells in solid tumors, we engineered M1EV with dual surface ligands, using hyaluronic acid (HA) to engage CD44 receptors on NK cells and cRGD peptides to target integrins overexpressed on cancer cells, generating a dual‐targeted construct, termed M1EV_HA/cRGD (Scheme 1) (Cho et al. 2016; Zhong et al. 2016; Rios et al. 2017; Ashrafizadeh et al. 2021; Park et al. 2012). This dual engineering enables M1EV_HA/cRGD to simultaneously engage NK and tumor cells, effectively bridging immune and cancer cells within the TME, and delivering the immunostimulatory cargos of M1EV. We hypothesized that this multifunctional dual‐targeting M1EV_HA/cRGD can enhance NK cell recruitment, activation, and cytotoxicity, thereby establishing a novel and potent immunotherapeutic strategy for solid tumors. We demonstrate that dual‐engineered M1EV_HA/cRGD promotes bridging between NK and breast cancer cells, enhancing NK cell clustering, activation, and cytotoxicity against breast tumor cells both *in vitro* and *in vivo*. Collectively, dual surface‐engineered M1EVs is a paradigm‐shifting immunotherapeutic approach that overcomes the dual challenges of TME immunosuppression and inefficient immune cell engagement. By bridging NK cells directly with tumor cells and remodeling the TME, this platform offers unprecedented potential for advanced solid tumor immunotherapy.

**SCHEME 1:**
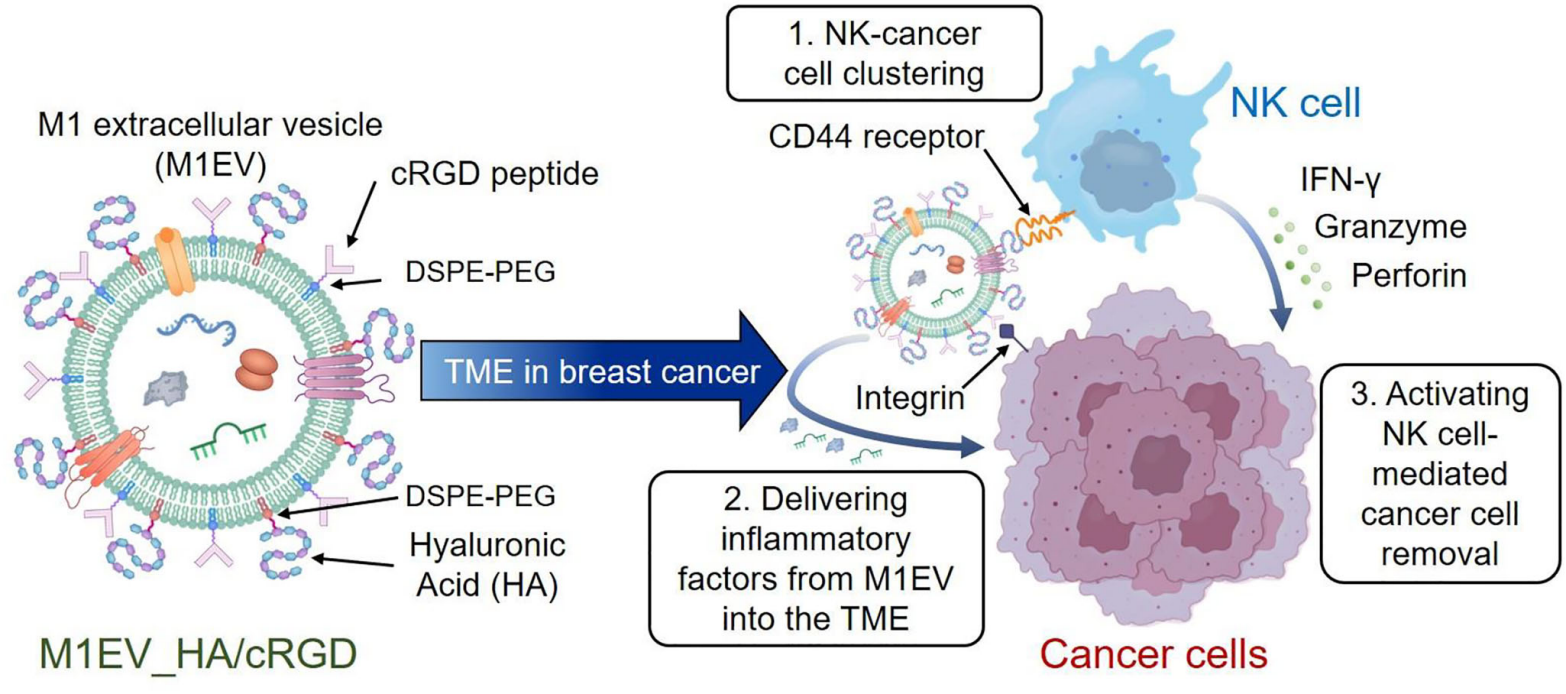
Schematic illustration of dual‐engineered M1EV_HA/cRGD for boosting NK cell mediated anti‐cancer effect. M1EV was surface modified with HA to bind CD44 receptors on NK cells and cRGD peptides to recognize integrins overexpressed on cancer cells. This dual‐targeting strategy allows M1EV_HA/cRGD to act as a biological bridge that connects and clusters NK and cancer cells within the TME, concurrently delivering inflammatory factors, such as cytokines and miRNAs to enhance NK cell activation and cytotoxicity against solid tumors. cRGD, cyclic RGD; IFN‐γ, interferon gamma; NK, natural killer; TME, tumor microenvironment.

## 2 Materials and methods

### 2.1 Cell Culture

Human breast cancer cells (MCF‐7) and human monocytes (THP‐1) were obtained from the Korean Cell Line Bank (Seoul, Republic of Korea). Human mammary epithelial cells (HMECs) were obtained from Lonza (Switzerland) and cultured in HuMEC Basal Serum‐Free Medium (Gibco, MA, USA). MCF‐7 and THP‐1 cells were cultured in RPMI‐1640 medium (Corning, NY, USA) supplemented with 10% (v/v) FBS (Gibco) and penicillin‐streptomycin (Gibco). Human NK cells (NK‐92 MI) were obtained from the American Type Culture Collection (ATCC, VA, USA) and cultured in Minimum Essential Medium Alpha (MEMα; Corning) supplemented with 12.5% (v/v) FBS (Gibco), 12.5% horse serum (Gibco), 0.2 mM inositol (Sigma‐Aldrich, MO, USA), 0.1 mM 𝛽‐mercaptoethanol (Sigma‐Aldrich), 0.02 mM folic acid (Sigma‐Aldrich), and penicillin‐streptomycin (Gibco). Murine breast carcinoma 4T1 cells were provided by Professor Byung‐Soo Kim (Seoul National University, Republic of Korea). The 4T1 cells were cultured in RPMI‐1640 medium (Corning) supplemented with 10% (v/v) FBS (Gibco) and penicillin‐streptomycin (Gibco). All cultures were maintained at 37°C with 5% CO_2_.

### 2.2 Induction and Characterization of M1‐Like Macrophages and Isolation of M1EVs

THP‐1 cells were treated with 200 ng/mL phorbol 12‐myristate 13‐acetate (PMA; Sigma‐Aldrich) for 24 h to induce pro‐inflammatory (M1)‐polarized macrophage. The 10% exosome‐depleted FBS (Gibco) containing RPMI medium was then replaced, and cells were further treated with 1,000 ng/mL of lipopolysaccharide (LPS; Sigma‐Aldrich) and 20 ng/mL of Human IFN‐gamma (IFN‐γ) recombinant protein (Gibco) (Lund et al. 2016; Lee and Sullivan 2001; Kang et al. 2019). To validate macrophage differentiation M1‐like polarization prior to M1EV isolation, THP‐1 monocytes, PMA‐differentiated macrophage‐like cells (M0‐like), and LPS/IFN‐γ‐stimulated M1‐like macrophages were analyzed by flow cytometry. Cells were stained with PE‐conjugated anti‐CD14 (Thermo Fisher Scientific, MA, USA), APC‐conjugated anti‐CD86 (Thermo Fisher Scientific), and Alexa Fluor 488‐conjugated anti‐CD206 antibodies (Thermo Fisher Scientific) according to the manufacturer's protocols. CD14, CD86, and CD206 were used as macrophage‐lineage, M1‐associated, and M2‐associated markers, respectively. Marker‐positive populations were determined using unstained controls under identical gating conditions.

For M1EV isolation, the conditioned medium from M1‐like macrophages was collected after 3 days, and cell debris was removed using centrifugation at 3,000 × *g* for 15 min, followed by filtration through a 0.2‐µm filter (GVS, Italy). The supernatant was concentrated using an Amicon Ultra‐15 filter (Millipore, MA, USA) with centrifugation at 5,000 × *g*. M1EVs were isolated from the concentrate using size‐exclusion chromatography (SEC), according to the manufacturer's protocol (Izon Science, New Zealand) (Kim et al. 2026).

### 2.3 Characterization of M1EV

The particle size and concentration of the M1EVs were measured using a Nanoparticle Tracking Analysis (NTA)—Nanosight NS300 system (Malvern, UK). For transmission electron microscopy (TEM) imaging, M1EVs were deposited onto Formvar carbon‐coated grids and incubated for 10 min, followed by rinsing with distilled water. The M1EVs were fixed in 2% paraformaldehyde, washed twice with phosphate buffered saline (PBS), and negatively stained with 2% uranyl acetate for 10 min. The grids were air‐dried for 15 min and imaged using a JEM‐1010 electron microscope (JEOL, Japan). The zeta potential and polydispersity index (PDI) of M1EVs were analyzed using a Zetasizer Nano ZS (Malvern).

### 2.4 Western Blotting of M1EV

M1EVs (5 × 10^9^ particles) were lysed in RIPA buffer and separated using SDS‐PAGE. Proteins were transferred to a NC membrane using transfer buffer. Membranes were blocked with 5% skim milk and incubated overnight at 4°C with primary antibodies: CD63 (MBL, Japan), CD81 (Abcam, UK), TSG101 (Abcam), and GRP78 (ABclonal, MA, USA). Anti‐mouse and anti‐rabbit IgG conjugated to horseradish peroxidase (Abcam; Cell Signaling Technology) were applied for 2 h at room temperature. The chemiluminescent signal was detected using ECL Prime Western Blotting Detection Reagent (Cytiva, MA, USA), and western blot images were captured on a ChemiDoc XRS+ System (Bio‐Rad, CA, USA).

### 2.5 qRT‐PCR Analysis

qRT‐PCR was performed using the StepOnePlus Real‐Time PCR System (Applied Biosystems, MA, USA). RNA extraction was performed using FavorPrep Tri‐RNA Reagent (FAVORGEN, Austria) and cDNA synthesis was carried out using the ReverTra Ace qPCR RT Master Mix (Toyobo, Japan). RT‐PCR was conducted using the THUNDERBIRD SYBR qPCR mix (Toyobo, Japan). The primer sequences are listed in Supplemental Table S1.

### 2.6 Evaluation of M1EV Cytotoxicity Against Cancer Cells

To evaluate the cytotoxic effects of M1EV on cancer cells, cell viability was assessed in the presence and absence of M1EV. MCF‐7 cells were seeded in 96‐well plates and incubated for 24 h, followed by treatment with M1EV at various concentrations ranging from 1 × 10^9^ to 1 × 10^10^ particles/mL for 96 h. Cell viability was measured using the WST‐1 assay (EZ‐Cytox, DoGENBio, Republic of Korea) following the manufacturer's instructions.

### 2.7 Small RNA Sequencing

To profile the miRNA content of M1EV, small RNA sequencing was performed (MACROGEN, Republic of Korea). Total RNA was extracted from isolated M1EV, and sequencing libraries were constructed using the SMARTer smRNA‐Seq kit (TaKara Bio, CA, USA). After RNA extraction and quality assessment, high‐throughput sequencing was performed using an Illumina platform. Data were normalized and analyzed for differential expression with a p‐value cutoff of <0.05. Gene Ontology (GO) and Kyoto Encyclopedia of Genes and Genomes (KEGG) pathway analyses were conducted to identify significant biological pathways and functions of differentially expressed genes.

### 2.8 Surface Engineering of M1EV with HA and cRGD Peptide

M1EV surface modification with HA for CD44 receptor targeting and cRGD peptide for integrin targeting was achieved using DSPE‐PEG conjugates synthesized via EDC/NHS chemistry. For DSPE‐PEG‐HA synthesis, HA (3‐5 kDa; 5 mg; Glentham Life Sciences, UK) was dissolved in 0.2 mL of buffer. DSPE‐PEG_2000_‐NH_2_ (5 mg; Biopharma PEG Scientific Inc., MA, USA) was separately dissolved in 0.5 mL of buffer. EDC (34 mg; Thermo Fisher Scientific) and sulfo‐NHS (12 mg; Thermo Fisher Scientific) were added to activate the carboxyl groups of HA, and the total reaction volume was adjusted to 2.0 mL with buffer. After 15 min of activation at room temperature, the DSPE‐PEG_2000_‐NH_2_ solution was added to the activated HA solution and reacted for 2 h at room temperature with gentle rotation. The reaction mixture was dialyzed against ultrapure water for three days using a dialysis membrane (Spectra/Por MWCO 2 kDa, Thermo Fisher Scientific) to remove unreacted reagents and subsequently lyophilized for at least three days to obtain DSPE‐PEG‐HA.

For DSPE‐PEG‐cRGD conjugation, DSPE‐PEG_2000_‐COOH (4.76 mg; Biopharma PEG Scientific Inc.) was dissolved in 1 mL of buffer, followed by the addition of EDC (6.8 mg) and sulfo‐NHS (2.4 mg) in buffer and incubation for 15 min. cRGD peptide (2.5 mg; MW: 947 Da; Peptron, Republic of Korea) dissolved in 1 mL of buffer was then added, and the final reaction mixture of 2 mL was reacted for 2 h at room temperature, dialyzed, and lyophilized, as described above. DSPE‐PEG_2000_‐COOH contains a single terminal carboxyl group, which was activated by EDC/sulfo‐NHS and reacted with the available amine groups of cRGD. Therefore, each DSPE‐PEG chain is expected to conjugate with no more than one cRGD molecule, although possible conjugation‐site heterogeneity within cRGD cannot be completely excluded.

Conjugation was confirmed via Fourier‐transform infrared (FT‐IR) spectroscopy (Bruker, MA, USA) and ^1^H NMR spectroscopy (JNM‐ECZ600R, JEOL, Japan). For semi‐quantitative estimation of conjugation degrees, the HA *N*‐acetyl proton signal at approximately 2.0 ppm, corresponding to three protons per HA repeating unit, and the DSPE methylene signal at approximately 1.25 ppm, corresponding to 56 internal methylene protons from the two stearoyl chains, were used as reference signals for DSPE‐PEG‐HA, and the apparent degree of substitution (DS) was calculated using: DSPE‐PEG/HA repeating unit ratio = (I_DSPE/56) / (I_HA/3). For DSPE‐PEG‐cRGD, the cRGD backbone α‐proton region at approximately 4.0‐4.4 ppm, corresponding to 12 protons from the cRGD backbone, and the DSPE methylene signal at approximately 1.0–1.5 ppm were used as reference signals, and the apparent conjugation ratio was calculated using: cRGD/DSPE‐PEG ratio = (I_cRGD/12)/(I_DSPE/56).

Critical micelle concentration (CMC) values of DSPE‐PEG‐HA and DSPE‐PEG‐cRGD were determined using pyrene as a hydrophobic fluorescent probe (Piñeiro et al. 2015; Wu et al. 2022). Briefly, DSPE‐PEG‐HA and DSPE‐PEG‐cRGD were prepared at serial concentrations in aqueous buffer and incubated with pyrene (Sigma‐Aldrich). After equilibration, fluorescence spectra were measured, and the pyrene intensity ratio of the third to first emission peak (I_3_/I_1_) was plotted against the logarithm of the DSPE‐PEG conjugate concentration. The CMC value was determined from the inflection point of the curve.

MALDI‐TOF MS analysis was performed using an autoflex maX system (Bruker Daltonics, Germany).

The apparent aqueous solubility/dispersibility of DSPE‐PEG‐HA and DSPE‐PEG‐cRGD was evaluated under the working conditions used for M1EV surface modification. DSPE‐PEG‐HA and DSPE‐PEG‐cRGD were prepared in aqueous buffer at 0.01 mg/mL and 0.02 mg/mL, respectively, by vortexing and gentle rotation; no visible precipitation was observed. Given that DSPE‐PEG‐HA and DSPE‐PEG‐cRGD are amphiphilic conjugates, these findings indicate apparent aqueous solubility/dispersibility rather than absolute molecular solubility. Surface‐engineered M1EVs (M1EV_HA/cRGD) were prepared by incubating M1EVs at a concentration of 1 × 10^11^ particles/mL with DSPE‐PEG‐HA and DSPE‐PEG‐cRGD at a 1:2 weight ratio at 37°C for 4 h, followed by ultrafiltration to remove unbound DSPE‐PEG conjugates.

### 2.9 Characterization of Surface Engineered M1EV_HA/cRGD

The particle size, concentration, zeta potential, and PDI of M1EV_HA/cRGD were measured and compared with those of M1EV. To quantify the amount of cRGD conjugated to M1EV, cRGD peptides were conjugated with fluorescein isothiocyanate (FITC), and the fluorescence intensity of cRGD‐FITC in the final M1EV_HA/cRGD product was measured using a Varioskan Flash microplate reader (Thermo Fisher Scientific). For fluorescence‐based assessment of HA incorporation, DSPE‐PEG‐HA was fluorescently labeled with Alexa Fluor 647‐Hydrazide (Thermo Fisher Scientific) through EDC/NHS‐mediated conjugation. Briefly, DSPE‐PEG‐HA was reacted with Alexa Fluor 647‐Hydrazide in the presence of EDC and NHS. The Alexa Fluor 647 labeled DSPE‐PEG‐HA (HA conjugate) was purified to remove unreacted dye and then used for M1EV surface modification together with DSPE‐PEG‐cRGD. After incubation and purification, the fluorescence signal associated with M1EV_HA/cRGD was measured using a Varioskan Flash microplate reader (Thermo Fisher Scientific). Control and unmodified M1EV groups were included to assess background fluorescence.

The retention time of conjugation stability on the surface of M1EV under high serum conditions was assessed by incubating M1EV_HA/cRGD with 50% exosome‐depleted FBS (Gibco) for 48 h. At 0, 6, 24, and 48 h, free HA/cRGD was removed using ultrafiltration (Pierce protein concentrators; Thermo Fisher Scientific). The EV particle count and fluorescence intensities of cRGD‐FITC and HA‐Alexa Fluor 647 were measured. The relative amount of each ligand was calculated as follows:

(Final amount of ligand/Initial amount of ligand) × 100.

### 2.10 Flow Cytometric Analysis of Enhanced Cellular Targeting by M1EV_HA/cRGD

The enhanced cellular targeting by M1EV_HA/cRGD was evaluated using flow cytometer (Beckman Coulter, CA, USA). M1EV_HA/cRGD was labeled with Vybrant DiI cell‐labeling solution (Invitrogen, MA, USA) and incubated with the target cells to assess uptake efficiency.

### 2.11 Confocal Microscopy for Analysis of M1EV Uptake

Cellular uptake of M1EVs was assessed using a confocal microscope (Zeiss, Germany). M1EVs were labeled with BODIPY FL C5‐Ceramide (Invitrogen) and subsequently incubated with the target cells. After 6 h of incubation, the cells were counterstained with Hoechst 33342 (Sigma‐Aldrich) to visualize the nuclei and with Alexa Fluor 594 Phalloidin (Thermo Fisher Scientific)‐stained F‐actin to delineate the cytoplasmic and cell‐boundary regions, and M1EV uptake was examined. For Z‐stack imaging, optical sections were acquired across the full cell depth to confirm intracellular localization of EV‐derived fluorescence. For receptor‐blocking assays, MCF‐7 cells were pretreated with an anti‐integrin αvβ5 antibody (Abcam) prior to incubation with fluorescently labeled EV formulations to assess the contribution of cRGD‐integrin interaction to tumor cell association. After antibody blocking, cells were incubated with fluorescently labeled M1EV or M1EV_HA/cRGD under the same conditions used for the cellular association assays.

### 2.12 Flow Cytometry and Western Blot Analysis of Receptor Expression

To compare the expression levels of receptors involved in M1EV_HA/cRGD‐mediated cell association, CD44 and integrin αvβ5 expression were analyzed in NK‐92 MI cells, MCF‐7 cells, and HMECs. For flow cytometric analysis, cells were harvested, washed, and incubated with antibodies against CD44 (Thermo Fisher Scientific) or integrin αvβ5 (Abcam) according to the manufacturer's instructions. For integrin αvβ5 detection, cells were further incubated with goat anti‐mouse IgG H&L Alexa Fluor 488 secondary antibody (Abcam). After washing, receptor‐associated fluorescence was measured using flow cytometer (Beckman Coulter). For western blot analysis, total cellular proteins were extracted and 20 µg of protein per sample was separated by SDS‐PAGE and transferred to a NC membrane. Membranes were blocked with 5% skim milk and incubated overnight at 4°C with primary antibodies against CD44 (Cell Signaling Technology), GAPDH (Cell Signaling Technology). Anti‐rabbit IgG conjugated to horseradish peroxidase (Cell Signaling Technology) were applied for 2 h at room temperature. The chemiluminescent signal was detected using ECL Prime Western Blotting Detection Reagent (Cytiva), and images were captured on a ChemiDoc XRS+ System (Bio‐Rad).

### 2.13 Western Blot Analysis of AKT Phosphorylation in NK‐92 MI Cells

To investigate the early signaling events underlying M1EV_HA/cRGD‐mediated NK cell activation, NK‐92 MI cells were co‐cultured with MCF‐7 cells at an E:T ratio of 1:2 in the presence or absence of M1EV or M1EV_HA/cRGD (5 × 10^9^ particles/mL). NK‐92 MI cells cultured alone without MCF‐7 cells or EV treatment were included as a negative control (NC). After 15 min of co‐culture, cells were harvested and lysed, and total protein was extracted. Proteins were separated by SDS‐PAGE and transferred to a NC membrane. For detection of total AKT and GAPDH, membranes were blocked with 5% skim milk and incubated overnight at 4°C with the corresponding primary antibodies (Cell Signaling Technology). For detection of phospho‐AKT, membranes were blocked with 5% bovine serum albumin (BSA) to prevent non‐specific binding of the phospho‐specific antibody, followed by overnight incubation at 4°C with an anti‐phospho‐AKT primary antibody (Cell Signaling Technology). Anti‐rabbit IgG conjugated to horseradish peroxidase (Cell Signaling Technology) was applied for 2 h at room temperature for all antibodies. The chemiluminescent signal was detected using ECL Prime Western Blotting Detection Reagent (Cytiva), and images were captured on a ChemiDoc XRS+ System (Bio‐Rad). Band intensities were quantified by densitometric analysis using ImageJ software, and the p‐AKT/AKT ratio was normalized to the CTRL group.

### 2.14 Evaluation of NK Cell Cytotoxicity Against Cancer Cells

To establish the optimal effector‐to‐target (E:T) ratio, cell viability was assessed in the absence of M1EV. MCF‐7 cells were seeded into 96‐well plates and incubated for 24 h, followed by treatment with NK‐92 MI cells at E:T ratios ranging from 1:10 to 10:1 for 24 h. Cell viability was measured using the WST‐1 assay (EZ‐Cytox, DoGENBio, Republic of Korea) following the manufacturer's instructions.

### 2.15 Assessment of M1EV_HA/cRGD Impact on Cancer Cell Lysis

Carboxyfluorescein succinimidyl ester (CFSE) dye that remains stable in viable cells but leaks upon cancer cell death, reducing fluorescence intensity. MCF‐7 cells were labeled with a CFSE—Cell labeling kit (Abcam) and co‐cultured with NK‐92 MI cells at an E:T ratio of 1:2 in the presence of M1EV_HA/cRGD. After 6 h, the cells were harvested and analyzed using flow cytometer (Beckman Coulter).

### 2.16 Flow Cytometry of E:T Cell Cluster Formation

MCF‐7 cells and HMECs were labeled with Vybrant DiD cell‐labeling solution (Invitrogen), and NK‐92 MI cells were labeled with Vybrant DiI cell‐labeling solution (Invitrogen). After labeling, the cells were washed and mixed at an E:T ratio of 1:2, followed by incubation at 0.5 and 2 h. M1EV_HA/cRGD was added at a concentration of 5 × 10^9^ particles/mL to assess its effect on cluster formation. Cell clusters were analyzed using flow cytometer (Beckman Coulter).

### 2.17 Enzyme‐linked Immunosorbent Assay (ELISA) for Cytokine and Cytotoxic Granule Protein Detection

ELISA was performed to assess the effect of M1EV_HA/cRGD on cytokine secretion and cytotoxic granule protein expression in NK cells. MCF‐7 cells were seeded in 24‐well plates and co‐cultured with NK cells at an E:T ratio of 1:2. The cells were treated with M1EV_HA/cRGD and incubated for 12 and 24 h. After incubation, the culture media were collected, and analyzed for IFN‐γ (Peprotech, NJ, USA), perforin (ABclonal), and granzyme B (GZB; Peprotech) levels using ELISA kits, according to the manufacturers’ protocols. A Varioskan Flash (Thermo Fisher Scientific) was used to measure absorbance.

### 2.18 *In Vivo* Evaluation Using an Orthotopic Breast Cancer Model

Female BALB/c mice (4‐6 weeks old) were obtained from DBL (Republic of Korea), and maintained under controlled temperature and humidity conditions with a 12‐h light‐dark cycle. All procedures complied with the ethical guidelines of the Incheon National University Animal Experiment Ethics Committee and were approved by the Committee (INU‐ANIM‐2024‐0035). The hair around nipple #4 was shaved using an electronic trimmer. The 4T1 cells (2 × 10^5^ cells/mouse) were injected into the mammary gland fat pad #4 (Paschall and Liu 2016; RAVAR et al. 2016). For the short‐term therapeutic study, treatment was initiated 7 days after tumor inoculation. M1EV formulations were administered via the tail vein every 3 days at a dose of 2 × 10^10^ particles/mL in a volume of 100 µL per injection. Tumor volume and body weight were monitored every 3 days, and tumor volumes were calculated using the following formula:

Tumor volume = [0.5 × (longest diameter) × (shortest diameter)^2^]

On Day 15, the tumors were excised, and weights were recorded. The tumors and organs (lungs, heart, liver, spleen, and kidneys) were harvested and fixed in 4% paraformaldehyde. The tumor tissue and organs were then embedded in paraffin and sectioned at 4 µm. Tumor tissue sections were subjected to hematoxylin and eosin (H&E) staining (Dako, Denmark), immunohistochemical staining using an anti‐Ki‐67 antibody (Cell Signaling Technology), and immunofluorescence staining using an anti‐CD56 antibody (Cell Signaling Technology), followed by optical (Leica, Germany) and fluorescence microscopy (Nikon, Japan).

For the extended therapeutic study, 4T1 tumor‐bearing BALB/c mice were randomly assigned to five groups: CTRL, M1EV, M1EV_HA, M1EV_cRGD, and M1EV_HA/cRGD. Each formulation was administered via the tail vein at the same dose (2 × 10^10^ particles/mL, 100 µL) on Days 0, 3, 6, 9, and 12 after treatment initiation. Tumor volume and body weight were monitored every 3 days until Day 30 to evaluate the sustained tumor growth‐suppressive effect of M1EV_HA/cRGD and to compare the long‐term efficacy of single‐ versus dual‐moiety formulations. On Day 30, tumors were excised, weighed, fixed in 4% paraformaldehyde, embedded in paraffin, and sectioned at 4 µm for histological analyses. Tumor cell apoptosis was further evaluated using a TUNEL Assay Kit (Cell Signaling Technology) according to the manufacturer's instructions.

### 2.19 Statistical Analysis

All data are presented as the mean ± standard deviation, with *n* ≥ 3. Statistical significance was determined using one‐way and two‐way ANOVA, with a *p* < 0.05 considered significant. Statistical analyses were performed using the GraphPad Prism 7.0 (GraphPad Software, CA, USA).

## 3 Results

### 3.1 Purification and Characterization of Pro‐inflammatory M1EVs

Pro‐inflammatory M1EVs were isolated and purified from M1‐polarized macrophages differentiated from THP‐1 monocytes. THP‐1 monocytes were first differentiated into macrophages using PMA, followed by polarization to M1 macrophages using LPS and IFN‐γ. Prior to M1EV isolation, the differentiation and polarization status of THP‐1‐derived macrophages was validated by flow cytometric analysis of CD14, CD86, and CD206 expression (Figure S1). The percentage of CD14‐positive cells was markedly increased in the PMA‐differentiated M0 macrophage‐like group, supporting the macrophage‐like differentiation of THP‐1 monocytes after PMA treatment. The M1‐like group showed a substantially higher percentage of CD86‐positive cells than both the monocyte and M0 groups, indicating enhanced M1‐associated activation following LPS/IFN‐γ stimulation. In contrast, CD206 expression remained low across all analyzed groups, confirming that the LPS/IFN‐γ‐treated cells did not acquire an M2‐like phenotype under the applied conditions. Together, these results confirm that THP‐1‐derived cells used for M1EV production were appropriately differentiated and polarized toward an M1‐like macrophage phenotype.

After culturing, the culture medium was collected, centrifuged to remove cell debris, and concentrated using ultrafiltration (UF, 100 K). M1EV were further purified using SEC and the highest concentrations were observed in fractions 6–8 (Figure 1A). Fractions 6–8 were pooled and analyzed using NTA, revealing an average particle size of 125.9 nm and a concentration of 5.76 × 10^10^ particles/mL (Figure 1B). Protein impurity analysis showed a purity level of 9.64 × 10^5^ particles/µg, confirming high purity (Figure 1C). The yield was 3.68 × 10^9^ particles/mL, comparable to the typical yields of mammalian cell‐derived EVs (Figure 1D). Characterization of M1EV was performed using western blotting (Figure 1E), which showed bands for CD63, CD81, and TSG101, known markers for mammalian cell‐derived EVs, whereas no band was detected for the negative marker, GRP78, confirming the high purity of M1EV.

**FIGURE 1:**
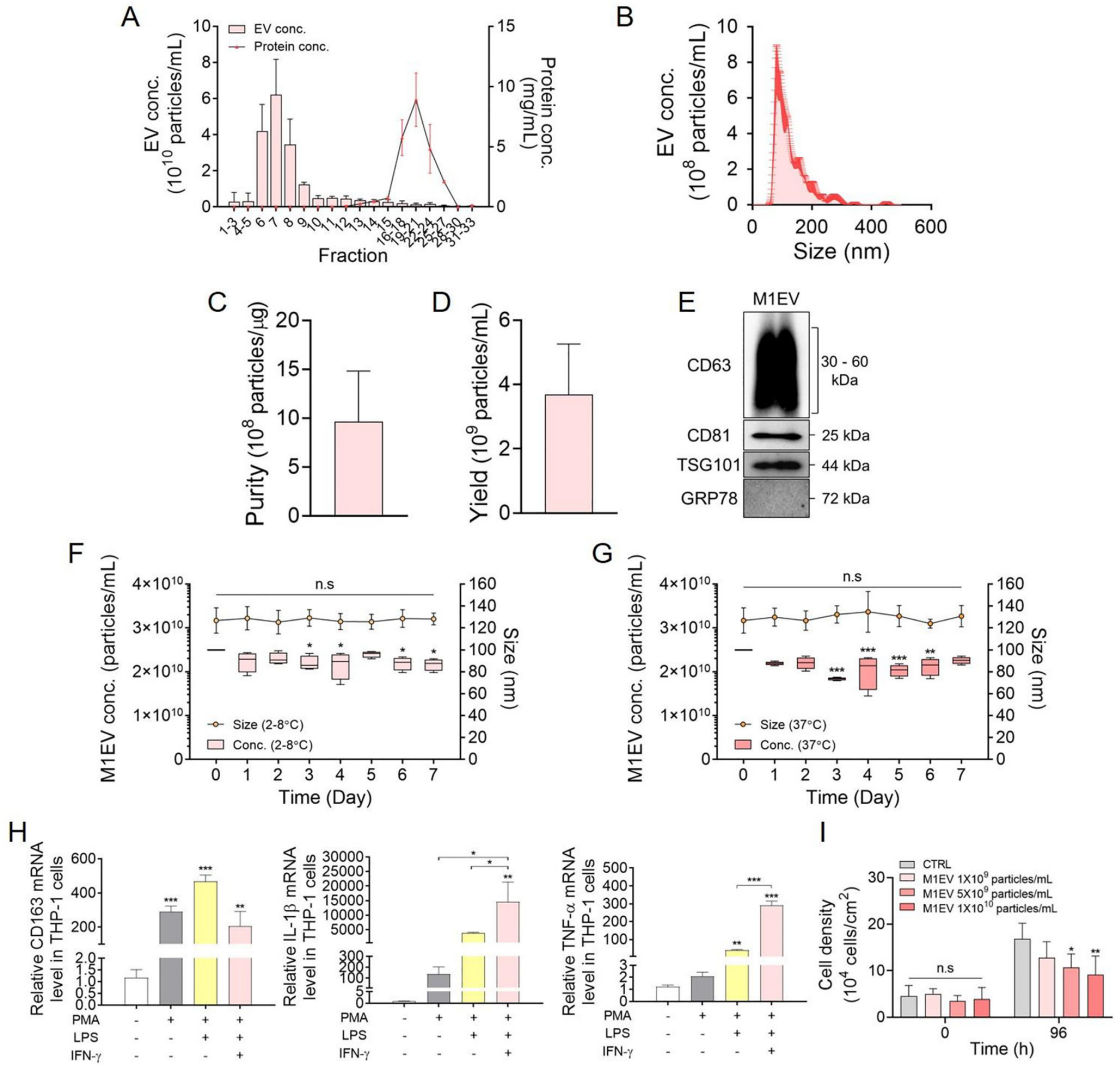
Isolation and characterization of M1EV. (A) Size‐exclusion chromatography profile showing highest M1EV concentrations in fractions 6–8. (B) Nanoparticle tracking analysis of pooled fractions 6–8. (C, D) Protein impurity (C) and yield analysis (D) of M1EV. (E) Western blot showing the presence of CD63, CD81, and TSG101, with no detection of GRP78, the negative EV marker. (F, G) Stability assessment of M1EVs at 2°C–8°C (F) and 37°C (G) in the presence of 50% serum over 7 days. (H) qRT‐PCR analysis of M1 macrophage inflammatory markers, indicating increased *IL‐1β* and *TNF‐α* mRNA levels in response to LPS and LPS+IFN‐γ treatments, confirming M1 polarization. (I) Anti‐cancer efficacy assessment in MCF‐7 cells treated with M1EV at 1 × 10^9^ to 1 × 10^10^ particles/mL. Data are presented as mean ± SD, with *n* ≥ 3. Statistical significance: *p < 0.05; **p < 0.01; ***p < 0.001; n.s., not significant. EV, extracellular vesicle; LPS, lipopolysaccharide; M1EV, M1‐polarized macrophage‐derived extracellular vesicle; PMA, phorbol 12‐myristate 13‐acetate.

The stability of M1EV was assessed using incubation at 2–8 and 37°C in the absence or presence of serum, monitoring changes in particle size and concentration over 7 days. First, while the concentration of M1EV slightly decreased, it remained high and stable at both temperatures in the absence of serum (Figure S2A, B). The M1EV stability was further observed through incubation at 2–8 and 37°C in the presence 50% serum. The concentration of M1EV under both temperatures remained relatively stable and no significant changes in EV size was observed (Figure 1F, G). This indicates that M1EVs remain stable under physiological conditions, suggesting that they can act effectively for extended periods when administered to humans. Furthermore, the development of tailored formulations of M1EVs could further enhance their storage and biostability.

To evaluate the biocompatibility of M1EV, its hemolytic properties were assessed using freshly collected blood samples. Owing to their small size and unique physicochemical characteristics, nanoparticles can potentially interact with red blood cells, leading to membrane disruption and subsequent hemolysis. The positive CTRL exhibited 92.8% hemolysis, whereas the M1EV group showed only 2.98% hemolysis (Figure S3). These results indicate that M1EVs are stable and highly biocompatible nanoplatforms suitable for biomedical applications.

### 3.2 Pro‐inflammatory and Anti‐cancer Function of M1EVs

To confirm the inflammatory characteristics of M1 macrophages from which M1EVs were isolated, differentiated M1 macrophages were analyzed for differentiation and polarization markers using qRT‐PCR (Figure 1H). CD163 was used as a marker to confirm monocyte‐to‐macrophage (M0) differentiation, while IL‐1β and TNF‐α were used to verify polarization to M1 macrophages. *CD163* mRNA levels indicated successful differentiation of monocytes into macrophages induced by PMA treatment. To further induce M1 polarization, cells were treated with either LPS or with both LPS and IFN‐γ (LPS+IFN‐γ). In the LPS group, *IL‐1β* mRNA expression increased by 3,952‐fold, whereas the LPS+IFN‐γ group showed an 14,584‐fold increase. *TNF‐α* mRNA expression increased by 40.6‐fold in the LPS group and 290.8‐fold in the LPS+IFN‐γ group. These results indicate that simultaneous treatment with LPS and IFN‐γ enhanced the inflammatory activation of macrophages more effectively than that using LPS alone. Additionally, IFN‐γ stimulation enhances the phagocytic activity of macrophages, thereby reinforcing their pro‐inflammatory functions, which are characteristic of M1 polarization (Lee and Sullivan 2001; Kang et al. 2019).

M1EV showed anti‐cancer activities via their encapsulated inflammatory cytokines and miRNAs derived from M1 macrophages (Bellingham et al. 2012; Choo et al. 2018; Pantazi et al. 2022; Kwak et al. 2022). To further validate this effect, M1EV was applied to MCF‐7 cells at concentrations ranging from 1 × 10^9^ to 1 × 10^10^ particles/mL, and the cell density was measured after 96 h using a cell counting assay (Figure 1I). Initially, the cells were seeded at a density of 5.0 × 10^4^ cells/cm^2^. After 96 h, the CTRL group proliferated to 1.69 × 10^5^ cells/cm^2^, whereas the M1EV‐treated groups reached only 1.28 × 10^5^, 1.08 × 10^5^, and 9.1 × 10^4^ cells/cm^2^ at 1 × 10^9^, 5 × 10^9^, and 1 × 10^10^ particles/mL, respectively. These results demonstrate that M1EVs inherently possess antitumor properties, highlighting their potential as a bioactive nanoplatform that can be further engineered into a potent immunomodulatory therapy.

### 3.3 Molecular Profiling of M1EVs for the Demonstration of Their Antitumor Potentials

EVs encapsulate various small RNAs, including miRNAs and Y RNAs, which can be delivered to recipient cells to modulate gene expression. To identify the molecules potentially responsible for the antitumor properties of M1EV, small RNA sequencing was performed. The RNA composition of M1EV showed a strong enrichment of small RNA reads, confirming the characteristic RNA profile of EVs. The small RNA fraction accounted for 7.39% of the total RNA reads from M1EV, reflecting the relative abundance of small RNA species, such as miRNA, tRNA, piRNA, and Y RNA (Figure 2A). Among these, miRNAs known to exhibit anti‐cancer properties were identified, comprising 0.024% of the known miRNAs and 1.627% of the novel miRNAs. Based on miRBase annotation, the identified miRNAs were classified into tumor suppressor, metastasis‐associated, inflammation‐associated, proliferation‐related, and unclassified groups. Notably, tumor suppressor miRNAs showed the highest expression levels (Figure 2B). In particular, tumor suppressor miRNAs, such as let‐7a‐5p, miR‐34c‐3p, and miR‐30e‐3p, and inflammation‐associated miRNAs, including miR‐21, miR‐155, and miR‐146, were abundantly detected, indicating their potential for delivery via M1EV.

**FIGURE 2:**
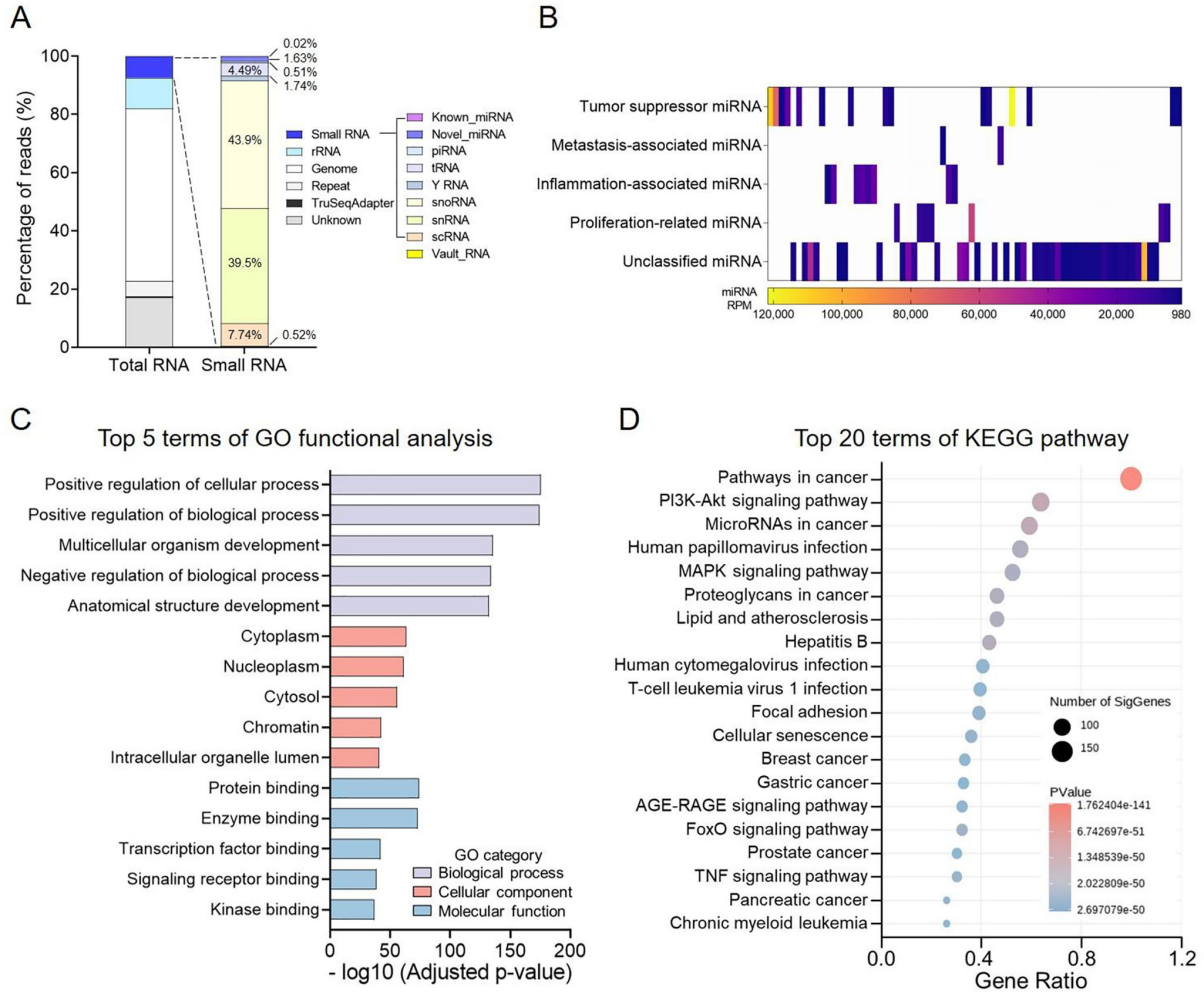
miRNA expression profiling and functional enrichment analysis of M1EV. (A) RNA composition of M1EV based on small RNA sequencing using the SMARTer smRNA‐Seq for Illumina platform. (B) Classification of identified miRNA according to miRBase annotation. (C) GO enrichment analysis of the top 5 functional categories associated with M1EV miRNA target genes. GO terms are categorized into biological process, cellular component, and molecular function. (D) KEGG pathway enrichment analysis of the top 20 signaling pathways targeted by M1EV‐derived miRNAs. GO, Gene Ontology; KEGG, Kyoto Encyclopedia of Genes and Genomes; M1EV, M1‐polarized macrophage‐derived extracellular vesicle.

To further elucidate the biological functions of M1EV‐derived miRNAs, GO and KEGG pathway enrichment analyses were performed. The top five GO terms were associated with biological processes, molecular functions, and cellular components (Figure 2C). M1EV miRNAs play key regulatory roles in cell growth, signal transduction, and developmental processes, particularly transcriptional regulation. KEGG pathway analysis of the top 20 enriched terms further demonstrated that M1EV miRNAs were associated with cancer pathways, cell differentiation, inflammatory responses, and intercellular immune signaling (Figure 2D). Specifically, enrichment in TGF‐β and Wnt signaling pathways suggests that M1EV may contribute to inflammation and modulation of the TME.

Collectively, these findings indicate that small RNAs, primarily miRNAs encapsulated within M1EV, can be functionally transferred to recipient cells and may play a crucial role in mediating the antitumor properties of M1EV.

### 3.4 Construction of the Dual‐targeting M1EV_HA/cRGD via Surface Engineering of M1EV with HA and cRGD Peptide

Positioning NK cells in close proximity to tumor cells is crucial for promoting NK cell‐mediated tumor cell elimination within the TME. We hypothesized that enhancing the association between NK and cancer cells would maximize anticancer efficacy by allowing NK cells to more efficiently recognize and interact with cancer cells. Therefore, to achieve this enhanced therapeutic effect, we aimed to establish a receptor‐assisted association platform between NK and cancer cells using surface‐engineered M1EVs. M1EVs were surface‐engineered with HA and cRGD peptides to promote receptor‐assisted interactions with NK cells and cancer cells, respectively. To conjugate HA and cRGD to M1EV, the carboxyl group of HA was linked to the amine group of DSPE‐PEG‐NH_2_, whereas the amine group of cRGD was linked to the carboxyl group of DSPE‐PEG‐COOH via an EDC/NHS‐mediated reaction (Figure 3A, D). HA with a molecular weight of 3–5 kDa was selected for DSPE‐PEG conjugation to minimize the intrinsic anti‐inflammatory or immunomodulatory effects associated with high‐molecular‐weight HA while retaining its role as a compact CD44‐binding ligand. The use of low‐molecular‐weight HA was also intended to reduce steric hindrance on the EV surface and maintain the nanoscale properties of M1EV_HA/cRGD after surface engineering (Bourguignon et al. 2011; Hoarau et al. 2022). Based on the molecular weight of the HA repeating disaccharide unit (∼400 Da), the 3–5 kDa HA used in this study was estimated to contain approximately 8–12 repeating disaccharide units per chain, providing multiple glucuronic acid‐derived carboxyl groups potentially available for EDC/NHS‐mediated conjugation. However, the actual conjugation degree was expected to be lower because of steric constraints and reaction‐efficiency limitations. After dialysis to remove the unreacted reagents, the conjugates were lyophilized to obtain DSPE‐PEG‐HA and DSPE‐PEG‐cRGD.

**FIGURE 3:**
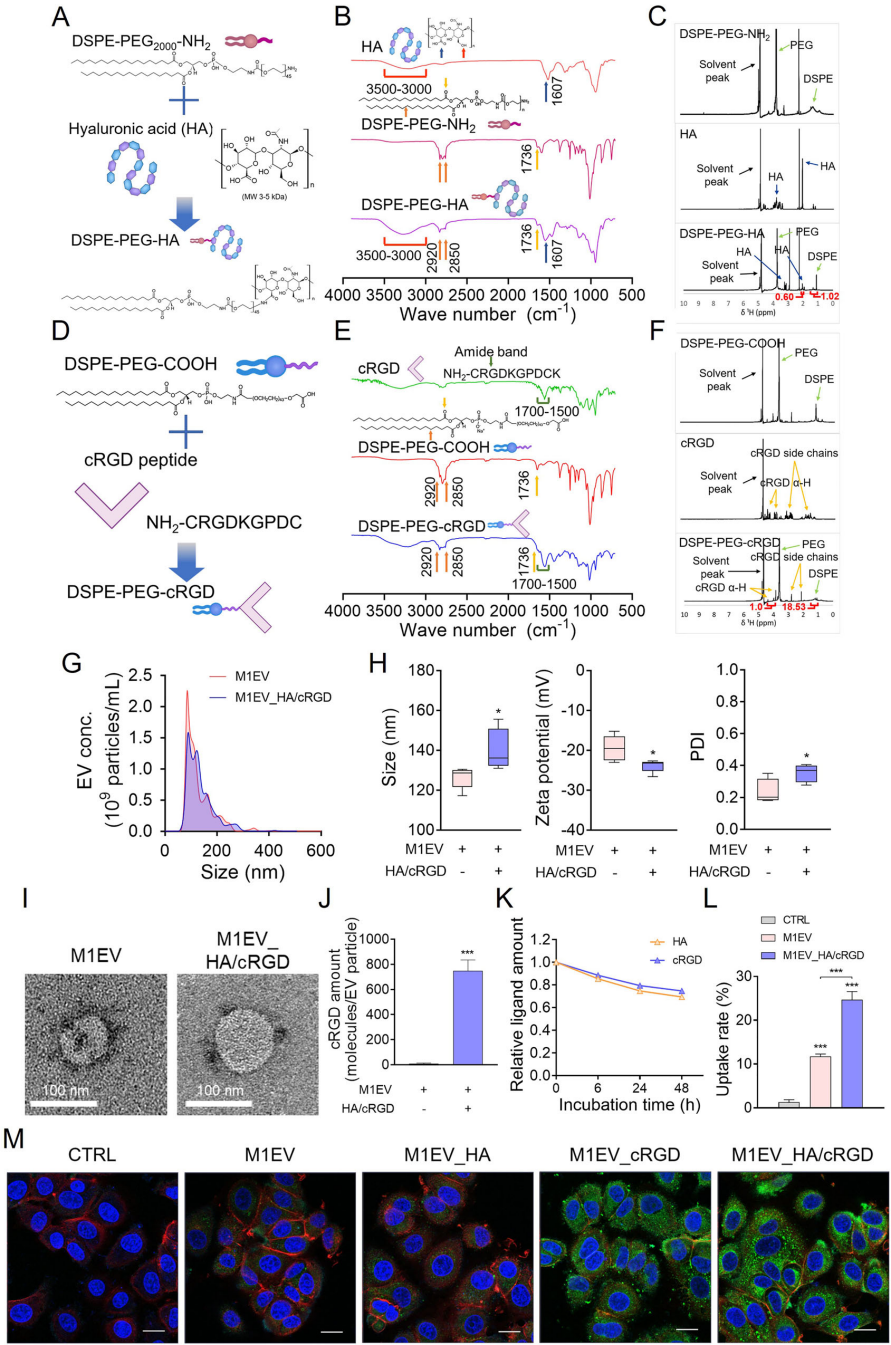
Surface engineering of M1EV for enhanced targeted therapy. (A) Schematic illustration of DSPE‐PEG‐HA conjugation to M1EV. (B) FT‐IR spectroscopy confirming the synthesis of DSPE‐PEG‐HA. Orange arrows indicate methylene groups of DSPE; yellow arrows, carbonyl groups of DSPE; red arrows, hydroxyl groups of HA and amine groups of DSPE; blue arrows, carboxylic groups of HA. (C) ^1^H NMR spectra of DSPE‐PEG‐NH_2_, HA, and DSPE‐PEG‐HA. Red brackets and values indicate the integration regions and corresponding integral values (I_HA = 0.60, I_DSPE = 1.02) used to calculate the apparent degree of substitution (DS). (D) Schematic illustration of DSPE‐PEG‐cRGD conjugation to M1EV. (E) FT‐IR spectroscopy confirming the synthesis of DSPE‐PEG‐cRGD. Orange arrows indicate methylene groups of DSPE; yellow arrows, carbonyl groups of DSPE; green arrows, amide band of cRGD peptide. (F) ^1^H NMR spectra of DSPE‐PEG‐COOH, cRGD, and DSPE‐PEG‐cRGD. Red brackets and values indicate the integration regions and corresponding integral values (I_cRGD = 1.00, I_DSPE = 18.53) used to calculate the apparent conjugation ratio. (G) Concentration and size assessment of M1EV_HA/cRGD after DSPE‐PEG‐HA and DSPE‐PEG‐cRGD conjugation. (H) Particle size analysis, zeta potential analysis, and PDI measurement. (I) Transmission electron microscopy images of M1EV_HA/cRGD; scale bars, 100 nm. (J) Quantification of cRGD molecules per M1EV particle using FITC‐labeled cRGD. Unmodified M1EVs were included as a negative control. (K) Retention time of M1EV_HA/cRGD. M1EV_HA/cRGD were cultured under high serum conditions for 48 h. The relative cRGD amount was assessed in comparison to the initial cRGD amount of M1EV_HA/cRGD. (L) Evaluation of targeting efficiency of M1EV_HA/cRGD targeting ability. Flow cytometric analysis of NK‐92 MI cells treated with DiI‐labeled M1EV and M1EV_HA/cRGD. (M) Confocal microscopy of MCF‐7 cells treated with BODIPY FL‐labeled M1EV, M1EV_HA, M1EV_cRGD, or M1EV_HA/cRGD. Blue, green, and red fluorescence represents the nuclei, BODIPY FL‐labeled EVs, and Alexa Fluor 594 Phalloidin‐stained F‐actin, respectively; scale bars, 50 µm. Data are presented as mean ± SD, with *n* ≥ 3. Statistical significance: *p < 0.05; ***p < 0.001. cRGD, cyclic RGD; EV, extracellular vesicle; M1EV, M1‐polarized macrophage‐derived extracellular vesicle; PDI, polydispersity index.

Although Gel Permeation Chromatography (GPC) analysis was attempted to determine the molecular weight distribution of DSPE‐PEG‐HA and DSPE‐PEG‐cRGD, reliable chromatograms could not be obtained under the tested conditions, likely due to the amphiphilic nature of the DSPE‐PEG‐HA and DSPE‐PEG‐cRGD conjugates and possible aggregation or column interaction. Therefore, the conjugation was instead verified using complementary analyses, including FT‐IR, ^1^H NMR, and fluorescence‐based assessment of HA and cRGD after M1EV surface modification.

Successful synthesis of both conjugates was confirmed by FT‐IR spectroscopy (Figure 3B, E) and further validated by ^1^H NMR analysis (Figure 3C, F). For DSPE‐PEG‐HA, semi‐quantitative integration of the HA *N*‐acetyl signal (I_HA = 0.60) and the DSPE methylene signal (I_DSPE = 1.02) yielded an apparent degree of substitution (DS) of approximately 9.1 mol% (Figure 3C). For DSPE‐PEG‐cRGD, integration of the cRGD backbone α‐proton signal (I_cRGD = 1.00) and the DSPE methylene signal (I_DSPE = 18.53) yielded an apparent conjugation ratio of approximately 25.2 mol% (Figure 3F).

MALDI‐TOF MS was performed to further characterize the DSPE‐PEG‐based conjugates (Figure S4). Comparison of DSPE‐PEG‐COOH and DSPE‐PEG‐cRGD showed an apparent mass increase of 926.8 ± 0.7 Da, supporting cRGD conjugation to DSPE‐PEG‐COOH alongside the FT‐IR and ^1^H NMR data (Figure 3E, F). Consistent with the known low ionization efficiency of polysaccharides in MALDI‐TOF MS (Hung et al. 2012), free HA and DSPE‐PEG‐HA did not yield assignable mass shifts under the tested condition.

The surface modification of M1EVs is likely mediated by lipid hydrophobic insertion of DSPE‐PEG‐based ligands. The hydrophobic DSPE lipid tail is expected to insert into or associate with the lipid bilayer of EV membranes through hydrophobic interactions, whereas the hydrophilic PEG chain extends toward the aqueous phase. Therefore, the HA and cRGD moieties conjugated to the distal end of PEG are expected to remain exposed on the outer surface of M1EV_HA/cRGD and available for receptor interaction. Because this process relies on non‐covalent lipid‐membrane anchoring rather than covalent modification of the EV membrane, it is expected to preserve EV structural integrity. The DSPE‐PEG‐HA:DSPE‐PEG‐cRGD input ratio of 1:2 was rationally selected based on the molecular size and steric properties of the two ligands. Because HA has a larger polymeric structure (3‐5 kDa) than the smaller cRGD peptide (MW: 947 Da), excessive HA density on the EV surface could sterically limit cRGD accessibility to integrins on tumor cells. Therefore, a higher relative input of DSPE‐PEG‐cRGD was used to help maintain cRGD availability while preserving HA‐mediated CD44 interaction. After incubation with M1EVs at 37°C for 4 h, unbound DSPE‐PEG‐HA and DSPE‐PEG‐cRGD were removed using ultrafiltration to obtain dual‐targeted M1EV (M1EV_HA/cRGD).

To further evaluate the colloidal self‐assembly behavior of the DSPE‐PEG‐based ligands, critical micelle concentration (CMC) analysis was performed using pyrene as a hydrophobic fluorescent probe. The CMC values of DSPE‐PEG‐HA and DSPE‐PEG‐cRGD were estimated to be 0.037 mg/mL and 0.022 mg/mL, respectively (Figure S5). Under the M1EV coating conditions used in this study, DSPE‐PEG‐HA was below its CMC, whereas DSPE‐PEG‐cRGD was close to but slightly below its CMC, suggesting that stable micelle formation was limited under the tested coating conditions. These results support the interpretation that DSPE‐PEG‐based ligands can dynamically associate with the EV lipid membrane during surface engineering.

The physicochemical properties of M1EV_HA/cRGD were characterized and compared with those of the unmodified M1EV. The particle concentration of M1EV_HA/cRGD (1.20 × 10^11^ particles/mL) remained comparable to that of unmodified M1EV (1.24 × 10^11^ particles/mL), indicating that surface conjugation did not affect EV yield (Figure 3G). The conjugation of HA and cRGD resulted in an increase in the average particle size from 126.4 to 139.7 nm (Figure 3H). Zeta potential decreased from −19.5 to −23.9 mV, likely due to the negative charge of HA in M1EV_HA/cRGD. A slight increase in PDI was observed, suggesting reduced particle uniformity owing to surface conjugation to M1EV. TEM analysis was performed to assess whether surface engineering altered the morphology of M1EVs. Compared with unmodified M1EVs, M1EV_HA/cRGD maintained a vesicle‐like structure without severe aggregation or structural disruption (Figure 3I), suggesting that vesicular morphology was largely preserved after surface modification. The amount of cRGD per M1EV particle was quantified using FITC‐labeled cRGD, showing 824.6 cRGD molecules per EV particle (Figure 3J). Unmodified M1EVs were included as a negative control to determine the background fluorescence signal. Compared with M1EVs, M1EV_HA/cRGD showed a markedly increased cRGD‐associated fluorescence signal, supporting successful cRGD incorporation during surface engineering. To further verify the incorporation of HA onto the engineered M1EVs, DSPE‐PEG‐HA was labeled with Alexa Fluor 647‐Hydrazide through EDC/NHS‐mediated conjugation and used for fluorescence‐based assessment after surface modification. Control and unmodified M1EV groups showed low background fluorescence signals, with mean fluorescence intensity values of 0.74 and 0.52, respectively. In contrast, M1EV_HA/cRGD showed a markedly increased HA‐associated fluorescence intensity of 11.08, supporting the incorporation of DSPE‐PEG‐HA onto the engineered M1EVs (Figure S6). Together with cRGD quantification, these results indicate that both HA and cRGD were retained in the dual‐ligand M1EV formulation, independent of the feed ratio used during synthesis, after the co‐incubation and purification process. The surface modification of M1EVs is likely mediated by the amphiphilic properties of DSPE‐PEG‐based ligands. The hydrophobic DSPE tail can associate with the lipid bilayer of EV membranes, whereas the PEG‐linked HA and cRGD moieties are exposed to the aqueous environment and available for receptor interaction. Because DSPE‐PEG‐HA and DSPE‐PEG‐cRGD share the same DSPE membrane‐associating domain, competitive insertion into the EV membrane may occur during co‐incubation. Therefore, the relative amount of each ligand on the final M1EV_HA/cRGD formulation may depend on the input ratio, concentration, incubation conditions, and colloidal state of the DSPE‐PEG derivatives. In this study, fluorescence‐based assessment of HA and cRGD in the final formulation showed that both ligands were retained after surface modification and purification.

To confirm the stability of HA/cRGD conjugated to the surface of M1EVs, its retention was assessed under high‐serum conditions (50% exosome‐depleted FBS). After incubation in high‐serum conditions, the relative amount of cRGD was maintained at 0.88 after 6 h, 0.79 after 24 h, and 0.75 after 48 h, and the relative amount of HA was maintained at 0.85 after 6 h, 0.75 after 24 h, and 0.69 after 48 h (Figure 3K). These results demonstrated that HA/cRGD, which can target NK and cancer cells, exhibited sufficient stability to remain conjugated to the surface of M1EVs.

### 3.5 M1EV_HA/CRGD Targeting Ability Toward NK and Cancer Cells

CD44, a plasma membrane glycoprotein, serves as the principal receptor for HA and plays a critical role in modulating cell adhesion, migration, proliferation, and downstream signaling cascades. Thus, the HA moiety on the M1EV_HA/cRGD surface facilitated selective interactions with NK cells through CD44 receptors. To investigate this targeting capability, NK‐92 MI cells were treated with DiI‐labeled M1EV and M1EV_HA/cRGD, and their targeting amounts were assessed. Flow cytometric analysis showed that 24.7% of M1EV_HA/cRGD was delivered to NK cells, compared with only 11.7% for unmodified M1EV, indicating the enhanced NK cell‐targeting capability of M1EV_HA/cRGD (Figure 3L).

Additionally, the breast cancer cell line, MCF‐7, expresses elevated levels of integrins that specifically recognize the RGD motif (Arg‐Gly‐Asp), enabling the selective binding of cRGD‐modified EVs (Taherian et al. 2011; Lee et al. 2014). To assess the individual contributions of each surface ligand to MCF‐7 cell association/internalization, MCF‐7 cells were treated with BODIPY FL‐labeled M1EV, M1EV_HA, M1EV_cRGD, or M1EV_HA/cRGD and examined by confocal microscopy (Figure 3M). The cRGD‐containing formulations, M1EV_cRGD and M1EV_HA/cRGD, exhibited substantially higher EV‐associated fluorescence than unmodified M1EV and M1EV_HA. M1EV_HA/cRGD showed a fluorescence intensity comparable to that of M1EV_cRGD, suggesting that cRGD‐integrin interactions are the dominant determinant of MCF‐7 cell association/internalization, whereas HA modification alone contributes minimally to uptake in this cell line. To further confirm the localization of EV‐derived fluorescence, double‐stained confocal imaging was performed using nuclear staining with Hoechst and cytoskeletal staining with F‐actin. EV‐derived fluorescence was predominantly distributed within F‐actin‐defined cytoplasmic regions, with negligible overlap with the nuclear compartment. Z‐stack confocal imaging further confirmed that M1EV_HA/cRGD‐derived fluorescence was detected across multiple optical sections of MCF‐7 cells, supporting intracellular/cell‐associated localization rather than surface‐only association (Figure S7). These findings indicate that M1EV_HA/cRGD preferentially associates with MCF‐7 cells in a cRGD‐integrin interaction‐dependent manner under the tested conditions.

To further examine whether the enhanced association of M1EV_HA/cRGD with MCF‐7 cells was related to cRGD‐integrin interaction, a receptor‐blocking assay was performed using an anti‐integrin αvβ5 antibody. Anti‐integrin blocking markedly reduced the M1EV_HA/cRGD‐derived fluorescence signal compared with the non‐blocking condition (Figure S8), supporting the involvement of cRGD‐integrin interaction in tumor cell association. Notably, the signal was not completely abolished following receptor blocking, indicating that additional EV‐cell interaction mechanisms may also contribute to cellular association. These data support a receptor‐associated component of M1EV_HA/cRGD tumor cell interaction, while precluding interpretation as an exclusively receptor‐dependent process.

To further clarify whether these receptor‐ligand interactions were cell‐type exclusive, CD44 and integrin αvβ5 expression were comparatively assessed in NK‐92 MI cells, MCF‐7 cells, and HMECs. Flow cytometric analysis demonstrated that CD44‐associated fluorescence was markedly higher in NK‐92 MI cells than in HMECs, and this finding was further confirmed by western blot analysis (Figure S9A, B). Similarly, integrin αvβ5‐associated fluorescence was significantly higher in MCF‐7 cells than in HMECs, as demonstrated by flow cytometric analysis and quantitative comparison (Figure 9C). These results indicate that CD44 and integrin αvβ5 are not exclusive markers restricted to a single cell type, but their relative expression levels differ substantially among the tested cell populations. Accordingly, M1EV_HA/cRGD is interpreted as a multivalent receptor‐assisted EV platform that preferentially enhances NK‐tumor cell association under the tested conditions, reflecting the higher relative expression of the corresponding receptors in NK‐92 MI and MCF‐7 cells compared with non‐malignant HMECs, rather than functioning as a strictly cell‐type‐exclusive targeting system.

### 3.6 Dual‐targeted M1EV_HA/cRGD Enhances NK Cell Clustering, Activation, and Anticancer Activity

NK cell‐mediated cytotoxicity is strongly influenced by the E:T ratio, because the frequency of NK‐tumor cell engagement determines the extent of immune activation and cancer cell killing. To assess the potential of engineered M1EV_HA/cRGD to enhance NK cell recognition and activation, the optimal E:T (NK‐92 MI:MCF‐7) ratio for cluster formation was first determined. MCF‐7 cells were treated with NK cells at various ratios ranging from 1:10 to 10:1, and cell viability was measured (Figure 4A). Overall, as the number of effector NK cells increased relative to that of the target cancer cells, a progressive decrease in MCF‐7 cell viability was observed, indicating enhanced NK cell‐mediated cytotoxicity in a ratio‐dependent manner. At an E:T ratio of 1:2, MCF‐7 cell viability decreased to 53.8% after 24 h, indicating significant cytotoxicity, while maintaining measurable differences among the experimental groups. Therefore, an E:T ratio of 1:2 was selected for further experiments because it provided measurable cytotoxicity while maintaining sufficient target cell survival to assess NK cell clustering and activation. M1EV_HA/cRGD was mixed with NK cells at a concentration of 5 × 10^9^ particles/mL and then applied to the MCF‐7 cells. After 6 h, breast cancer cell viability in the presence of NK cells without M1EV_HA/cRGD decreased to 83.2%, whereas breast cancer cells treated with both NK cells and M1EV_HA/cRGD showed a further decrease to 68.3% (Figure 4B). At 24 h, cell viability further decreased to 34.5% in the NK cell‐only control group and to 28.7% in the NK cell‐ and M1EV_HA/cRGD‐treated group. These results suggest that M1EV_HA/cRGD moderately enhanced early NK cell‐mediated cytotoxicity under the tested co‐culture conditions, although the difference between groups became less pronounced at 24 h, when NK cell‐mediated killing was already substantial in the control group.

**FIGURE 4:**
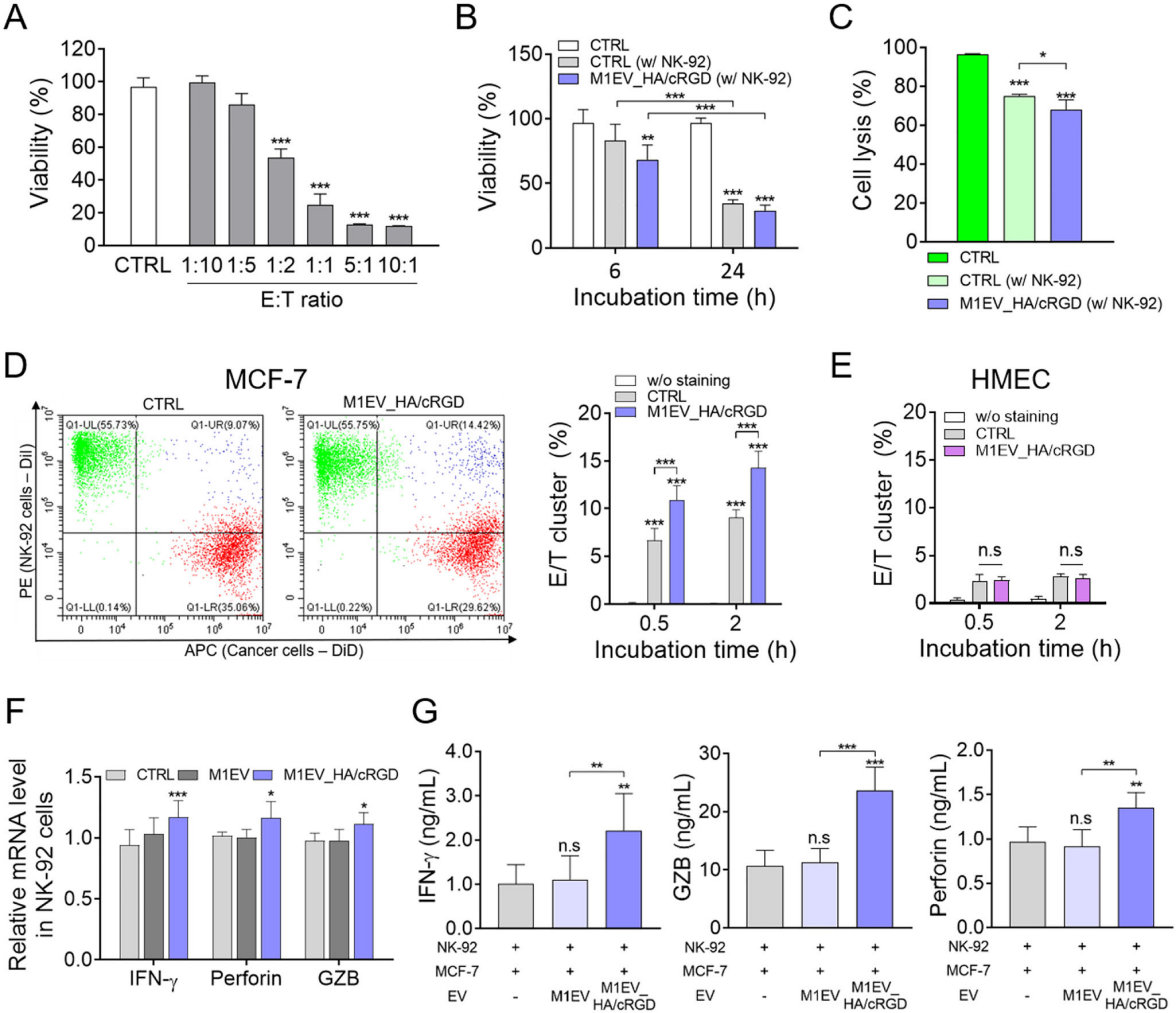
Evaluation of cytotoxicity and clustering enhancement by M1EV_HA/cRGD. (A) Cell viability analysis of MCF‐7 cells treated with NK cells at various E:T ratios (1:10–10:1). (B) WST‐1 assay showing MCF‐7 cell viability with NK cells and M1EV_HA/cRGD after 6 and 24 h. (C) Cancer cell lysis confirmed by CFSE staining of MCF‐7 cells. (D) Flow cytometric analysis of cluster formation between NK and MCF‐7 cells. (E) Flow cytometric analysis of cluster formation between NK and HMEC. (F) qRT‐PCR analysis of NK cell activation markers. (G) ELISA of NK cell activation markers. Data are presented as mean ± SD, with *n* ≥ 3. Statistical significance: *p < 0.05; **p < 0.01; ***p < 0.001; n.s., not significant. cRGD, cyclic RGD; E:T, effector‐to‐target; EV, extracellular vesicle; GZB, granzyme B; M1EV, M1‐polarized macrophage‐derived extracellular vesicle; NK, natural killer.

To confirm cancer cell lysis induced by M1EV_HA/cRGD, MCF‐7 cells were stained with CFSE (Yang et al. 2024). In the untreated CTRL group (MCF‐7 cells only), the CFSE fluorescence intensity remained at 96.5%, indicating high cell viability (Figure 4C). Following treatment with NK cells alone, the fluorescence intensity decreased to 75.1% after 6 h, indicating NK cell‐mediated cytotoxicity in breast cancer cells. Notably, when MCF‐7 cells were cotreated with NK cells and M1EV_HA/cRGD, the fluorescence intensity decreased further to 68.0%, confirming that M1EV_HA/cRGD effectively enhanced NK cell‐mediated cancer cell lysis. Together, these results suggest that M1EV_HA/cRGD facilitates NK‐tumor cell association and may contribute to enhanced NK‐mediated cytotoxic responses under the tested conditions, although increased clustering alone may not fully account for the extent of downstream tumor killing.

The clustering ratios of NK and MCF‐7 cells were evaluated at an E:T ratio of 1:2. MCF‐7 cells were labeled with DiD, NK cells were labeled with DiI, and cluster formation was analyzed using flow cytometer (Figure 4D). After 30 min, spontaneous clustering between NK and MCF‐7 cells in the absence of M1EV_HA/cRGD (CTRL) was observed at 6.7%, whereas treatment with 5 × 10^9^ particles/mL of M1EV_HA/cRGD increased clustering to 10.9%. After 2 h, the control group exhibited a slight increase in clustering to 9.1%, whereas the M1EV_HA/cRGD‐treated group exhibited a further increase to 14.2%. We further demonstrated that the clustering activity of M1EV_HA/cRGD was specific to NK and cancer cells. In contrast to the increased NK‐MCF‐7 clustering observed following M1EV_HA/cRGD treatment, NK‐HMEC clustering remained low and was not substantially increased by M1EV_HA/cRGD treatment (Figure 4E). Given that CD44 expression was higher in NK cells than in HMECs and integrin αvβ5 expression was stronger in MCF‐7 cells than in HMECs (Figure S9), these results indicate that M1EV_HA/cRGD‐mediated clustering is not driven by HA‐CD44 interaction alone. Rather, efficient NK‐tumor cell clustering appears to require the cooperative contribution of both HA‐CD44 and cRGD‐integrin interactions, together with a sufficient level of the corresponding receptors on the target cell population. Therefore, M1EV_HA/cRGD preferentially enhanced NK‐tumor cell association under the tested conditions.

To investigate the activation and cytotoxic function of NK cells enhanced by M1EV_HA/cRGD, which promotes NK cell recognition, qRT‐PCR and ELISA were performed to measure the key cytotoxic factors. Upon recognizing cancer cells, NK cells release the cytokine IFN‐γ, along with cytotoxic granules, such as perforin and GZB, to induce cancer cell death (Trapani and Smyth 2002). NK (effector) and MCF‐7 (target) cells were co‐cultured at a 1:2 ratio, and M1EV_HA/cRGD was added at a concentration of 5 × 10^9^ particles/mL. Results showed an increase in *IFN‐γ* mRNA levels from 0.94 to 1.17, *perforin* mRNA levels from 1.12 to 1.17, and *GZB* mRNA levels from 0.98 to 1.11 (Figure 4F). ELISA of the cell culture supernatant revealed an elevated secretion of cytokines and cytotoxic granules following treatment with M1EV and M1EV_HA/cRGD (Figure 4G). In the CTRL group (NK and MCF‐7 co‐culture without any EV treatment), IFN‐γ, perforin, and GZB levels were measured at 1.01, 0.97, and 10.70 ng/mL, respectively. Treatment with M1EV slightly increased these levels to 1.11, 0.92, and 11.23 ng/mL, while M1EV_HA/cRGD treatment further enhanced secretion to 2.21, 1.35, and 23.67 ng/mL, respectively. These results indicated that M1EV_HA/cRGD enhanced NK cell activation and cytotoxic functions at both the transcriptional and protein levels.

To further investigate the early signaling events underlying M1EV_HA/cRGD‐mediated NK cell activation, the phosphorylation status of AKT was assessed in NK‐92 MI cells as early as 15 min after co‐culture initiation with MCF‐7 cells in the presence or absence of M1EV or M1EV_HA/cRGD (Figure S10A and S10B). The p‐AKT/AKT ratio was significantly increased in the M1EV_HA/cRGD‐treated group compared with the CTRL and M1EV groups at this early timepoint, indicating rapid AKT pathway activation under the tested conditions. These results suggest that M1EV_HA/cRGD promotes NK cell activation at least in part through early intracellular signaling pathway engagement, consistent with the enhanced effector molecule secretion observed in Figure 4G. Although the precise upstream trigger of AKT activation remains to be fully elucidated, these findings support the interpretation that M1EV_HA/cRGD enhances NK cell immunotherapeutic activity through a combination of receptor‐assisted cell association, immune synapse facilitation, and early signaling pathway modulation.

Collectively, M1EV_HA/cRGD promoted NK‐tumor cell association and supported NK functional activation, as evidenced by increased effector molecule secretion and a moderate enhancement of early NK‐mediated tumor cell lysis. These findings suggest that the dual‐ligand M1EV platform may enhance NK cell immunotherapeutic activity through a combination of multivalent receptor‐assisted cell association and EV‐mediated immunomodulatory effects, rather than through physical clustering alone.

### 3.7 Short‐term *in Vivo* Assessment of the Antitumor Efficacy of M1EV_HA/cRGD

Our *in vitro* experiments demonstrated that M1EV_HA/cRGD exerts potent anticancer effects through multiple synergistic mechanisms. Specifically, inherent components of M1EV contribute directly to cancer cell death. Furthermore, their dual‐targeting capability significantly promotes effective clustering between NK and cancer cells, thereby amplifying NK cell‐mediated cytotoxicity. Based on these mechanistic *in vitro* findings, we used an *in vivo* mouse model to demonstrate how the dual targeting and immunostimulatory properties of M1EV_HA/cRGD translate into potent antitumor activity *in vivo*.

The antitumor efficacy of the dual‐target M1EV_HA/cRGD was evaluated in a 4T1 orthotopic mouse cancer model established in BALB/c mice. The CTRL group received PBS (without EVs) every 3 days via intravenous injection. M1EV and M1EV_HA/cRGD were administered to 4T1 tumor‐bearing mice via an intravenous injection of 2 × 10^10^ particles every 3 days (Figure 5A). Mouse body weight and tumor volume were monitored every 3 days and tumors were excised on Day 15. By Day 15, the body weights of the CTRL‐, M1EV‐, and M1EV_HA/cRGD‐treated groups had increased to 19.8, 20.6, and 20.3 g, respectively (Figure 5B). The tumor volume in the CTRL group substantially increased to 682.5% from Day 0 to 15 (Figure 5C). In contrast, the tumor volume increased by only 346.5 and 204.4% in the M1EV‐ and M1EV_HA/cRGD‐treated groups, respectively, indicating effective tumor growth suppression by M1EV‐based treatment. Importantly, M1EV_HA/cRGD showed a significantly stronger tumor‐suppressive effect than non‐engineered M1EV, supporting the enhanced *in vivo* antitumor activity of the dual ligand engineered formulation.

**FIGURE 5:**
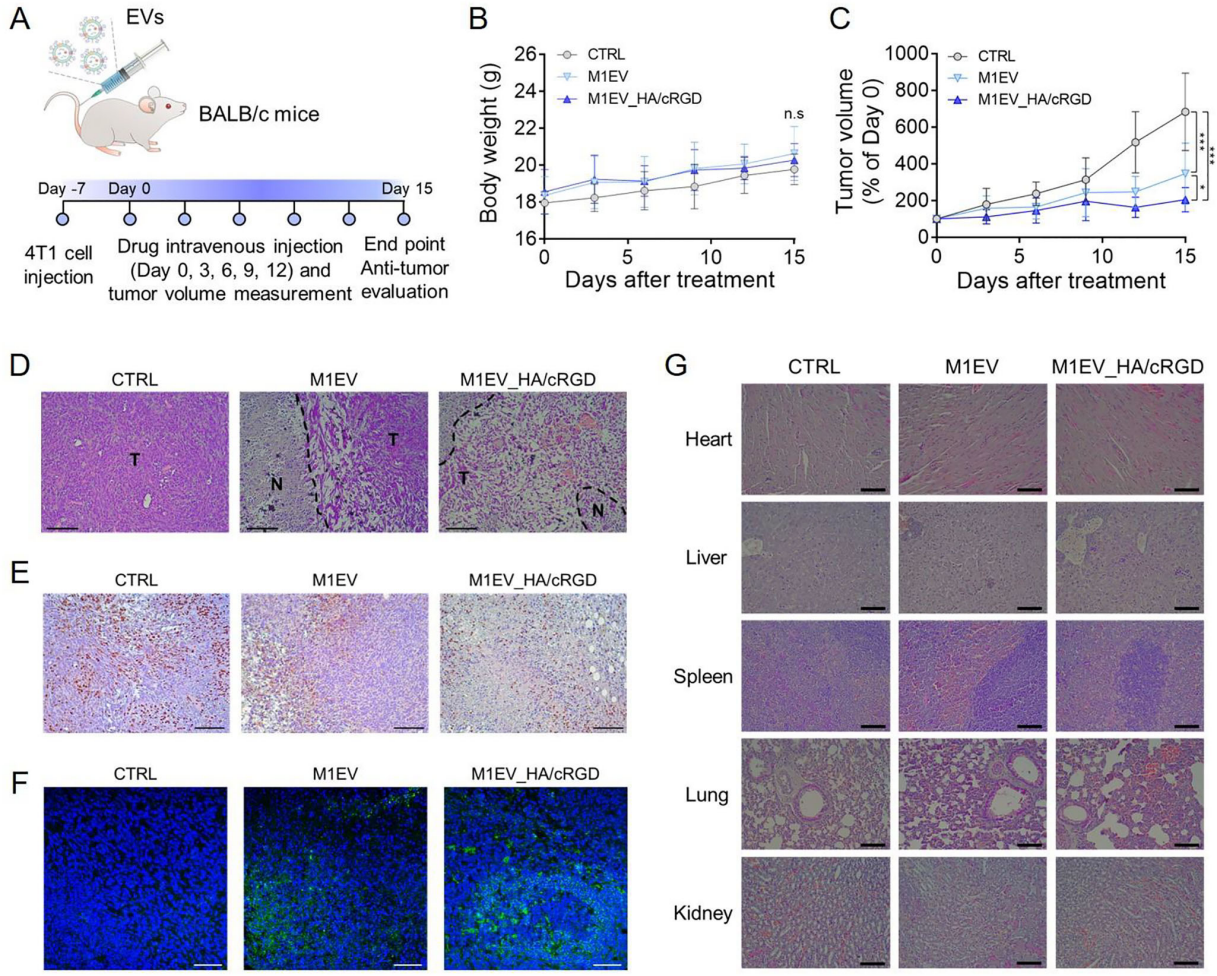
Antitumor efficacy of M1EV and M1EV_HA/cRGD in a 4T1 orthotopic mouse cancer model. (A) Schematic illustration of the development of a 4T1 orthotopic mouse cancer model. (B) Body weight changes by Day 15. (C) Tumor growth profiles in CTRL (PBS), M1EV (2 × 10^10^ particles/mL), and M1EV_HA/cRGD (2 × 10^10^ particles/mL) treated groups over Day 15. (D) H&E staining of tumor tissue after 15 days of treatment. The boundary between the tumor (T) and the necrotic region (N) is outlined with a dotted line in the images; scale bar 100 µm. (E, F) IHC (Ki‐67) staining and IF (CD56) of tumor tissue after 15 days of treatment. (G) Biocompatibility assessment of M1EV_HA/cRGD. Histological analysis of major organs (heart, liver, spleen, lungs, and kidneys) on Day 15 for biocompatibility evaluation of M1EV_HA/cRGD, with H&E staining results showing scale bars, 100 µm. Data are presented as mean ± SD, with *n* ≥ 3. Statistical significance: *p < 0.05; ***p < 0.001; n.s., not significant. cRGD, cyclic RGD; H&E, hematoxylin and eosin; M1EV, M1‐polarized macrophage‐derived extracellular vesicle.

Histological analysis of tumor sections stained with H&E revealed that tumor regions were predominant in the CTRL group (Figure 5D). However, tumor tissue from mice treated with M1EV revealed the presence of necrotic regions, which supports the previously described anticancer function of M1EV. Notably, the M1EV_HA/cRGD‐treated group exhibited the most extensive necrosis within the tumor mass, indicating enhanced tumor suppression. Immunohistochemical analysis revealed that Ki‐67, a cell proliferation marker, showed high expression in the CTRL group. Although Ki‐67 expression was reduced in the M1EV‐treated group, the M1EV_HA/cRGD‐treated group showed the most significant suppression of cell proliferation (Figure 5E). Furthermore, immunofluorescence staining for CD56, a representative marker of NK cells, revealed enhanced NK cell infiltration in the treatment group (Figure 5F). The CD56‐positive regions were limited to the CTRL group, moderately increased in the M1EV‐treated group, and most abundant in the M1EV_HA/cRGD group. Quantitative image analysis further supported these observations. Ki‐67‐positive area was significantly reduced from 13.84% in the CTRL group to 5.52% in the M1EV group and 1.46% in the M1EV_HA/cRGD group (Figure S11A). CD56‐positive area increased significantly from 12.80% in the CTRL group to 19.90% in the M1EV group and 41.53% in the M1EV_HA/cRGD group (Figure S11B), consistent with the enhanced NK cell infiltration and reduced tumor cell proliferation observed across the treatment groups.

These *in vivo* findings are consistent with our *in vitro* results showing that HA/cRGD modification facilitates receptor‐assisted NK‐tumor cell interactions. In addition, the reduced Ki‐67‐positive area and increased CD56‐positive area in M1EV_HA/cRGD‐treated tumors suggest that the enhanced tumor growth suppression may be associated with reduced tumor cell proliferation and increased NK cell accumulation in the TME. Collectively, these results support the enhanced *in vivo* antitumor activity of M1EV_HA/cRGD compared with non‐engineered M1EV.

The biocompatibility of M1EV_HA/cRGD was evaluated through intravenous injection in a 4T1 orthotopic cancer model, followed by the collection of major organs for histological analysis (Figure 5G). H&E staining of the heart, liver, spleen, lungs, and kidneys revealed no observable adverse effects in either the M1EV‐ or M1EV_HA/cRGD‐treated groups compared with those in the CTRL group. These findings underscore the high biocompatibility of M1EV_HA/cRGD, indicating its potential for clinical applications with minimal risk of systemic toxicity or adverse side effects. Consistent with previous studies, M1EV alone exhibits antitumor effects, whereas M1EV_HA/cRGD developed in the study demonstrated enhanced antitumor efficacy over M1EV, indicating its potential as an effective anticancer agent with reduced side effects. These results further underscore that surface‐engineered EVs can act as versatile and biocompatible immunotherapeutic platforms, integrating tumor targeting, immune activation, and reduced systemic toxicity, a crucial step toward next‐generation precision immunotherapy.

### 3.8 Long‐term Antitumor Efficacy of Dual‐targeted M1EVs Relative to Single‐moiety Controls

The *in vitro* and Day 15 *in vivo* findings demonstrated that dual‐targeted M1EV_HA/cRGD enhances NK cell‐mediated cytotoxicity and tumor suppression compared with unmodified M1EV. However, these early‐stage analyses alone could not fully capture the sequential immunological events occurring within the TME, nor could they isolate the individual contributions of the HA and cRGD moieties to the observed therapeutic effect. To address this, we extended the *in vivo* observation period to Day 30 and incorporated single‐moiety control formulations, M1EV_HA and M1EV_cRGD, in addition to CTRL, M1EV, and M1EV_HA/cRGD, enabling a direct comparison of single‐ versus dual‐targeting strategies under long‐term treatment conditions (Figure 6A, B). Throughout the 30‐day treatment period, body weights remained comparable across all five groups (CTRL: 21.99 g; M1EV: 21.39 g; M1EV_HA: 22.31 g; M1EV_cRGD: 21.94 g; M1EV_HA/cRGD: 21.36 g) (Figure 6C), with no apparent weight loss observed in any of the M1EV‐treated groups relative to CTRL. In contrast, tumor volume measurements revealed clear differences in therapeutic efficacy among the treatment groups (Figure 6D). By Day 30, tumor volume in the CTRL group increased to 2384.0% relative to Day 0, whereas the M1EV‐, M1EV_HA‐, and M1EV_cRGD‐treated groups reached 1408.5%, 1477.6%, and 1553.1%, respectively, and the M1EV_HA/cRGD‐treated group showed the lowest tumor volume among all groups at 921.4%. Statistical significance relative to CTRL emerged from Day 27 onward and was sustained through Day 30, with M1EV_HA/cRGD showing the strongest and most consistent significance among all groups at both time points (Table S3). The Day 15 short‐term study (Figure 5C) and the Day 30 extended study (Figure 6D) represent two independent *in vivo* experiments. To determine whether dual‐surface engineering provides greater antitumor efficacy than single‐ligand modification, tumor tissues collected at Day 30 were further examined by H&E, Ki‐67, and TUNEL staining. H&E staining revealed that tumor regions were largely intact in the CTRL group, whereas necrotic areas became progressively more evident in the M1EV‐, M1EV_HA‐, and M1EV_cRGD‐treated groups (Figure 6E). Among all groups, the M1EV_HA/cRGD‐treated group displayed the most extensive necrotic regions within the tumor mass, consistent with its superior tumor growth‐suppressive effect. Immunohistochemical staining for Ki‐67 showed that the CTRL group retained a high density of proliferating tumor cells, while Ki‐67 expression was visibly reduced across all M1EV‐treated groups, with the most pronounced reduction observed in the M1EV_HA/cRGD group (Figure 6F). TUNEL staining was additionally performed to confirm tumor cell apoptosis (Figure 6G). TUNEL‐positive signals were minimal in the CTRL group, modestly increased in the M1EV‐, M1EV_HA‐, and M1EV_cRGD‐treated groups, and most prominent in the M1EV_HA/cRGD‐treated group. Quantitative image analysis confirmed a clear histological advantage for the dual‐engineered formulation: Ki‐67‐positive area was significantly lower in M1EV_HA/cRGD than in M1EV (0.53% vs. 6.00%), and TUNEL‐positive area in M1EV_HA/cRGD (10.93%) was significantly higher than in each of the other treatment groups (M1EV: 1.70%; M1EV_HA: 2.03%; M1EV_cRGD: 2.80%) (Figure S12), demonstrating that dual‐surface modification promoted significantly greater tumor cell apoptosis than either single‐ligand formulation.

**FIGURE 6:**
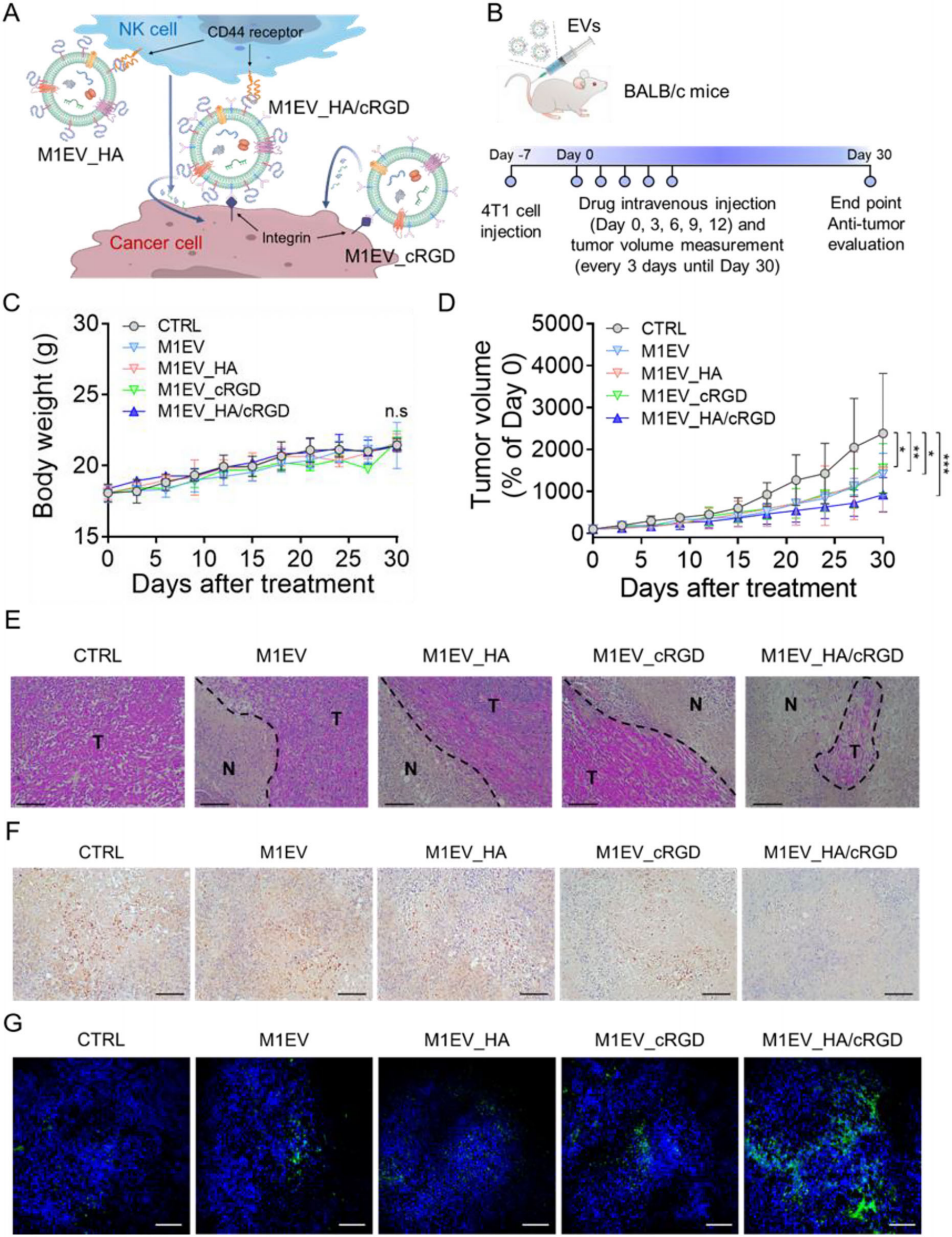
Long‐term antitumor efficacy of M1EV formulations with single‐ and dual‐moiety surface modifications in a 4T1 orthotopic mouse cancer model. (A) Schematic illustration of the proposed targeting mechanisms of M1EV_HA, M1EV_cRGD, and M1EV_HA/cRGD, depicting HA‐mediated binding to CD44 receptors on NK cells and cRGD‐mediated binding to integrins on cancer cells. (B) Schematic illustration of the experimental timeline for the long‐term *in vivo* antitumor study. (C) Body weight changes over 30 days across all treatment groups. (D) Tumor growth profiles in CTRL (PBS), M1EV, M1EV_HA, M1EV_cRGD, and M1EV_HA/cRGD (2 × 10^10^ particles/mL) treated groups monitored until Day 30. (E) H&E staining of tumor tissue collected at Day 30. The boundary between the tumor (T) and the necrotic region (N) is outlined with a dotted line; scale bar, 100 µm. (F) IHC (Ki‐67) staining of tumor tissue collected at Day 30; scale bar, 100 µm. (G) TUNEL staining of tumor tissue collected at Day 30; scale bars, 100 µm. Data are presented as mean ± SD, with *n* ≥ 3. Statistical significance: *p < 0.05; **p < 0.01; ***p < 0.001; n.s., not significant. cRGD, cyclic RGD; H&E, hematoxylin and eosin; M1EV, M1‐polarized macrophage‐derived extracellular vesicle; TUNEL, terminal deoxynucleotidyl transferase dUTP nick end labeling.

Collectively, these long‐term findings, encompassing tumor growth kinetics, histological necrosis, proliferation marker expression, and apoptotic cell death, demonstrate that the antitumor benefit of dual‐surface engineering with both HA and cRGD is sustained and becomes increasingly evident at the histological level with extended treatment duration. Together with the NK cell infiltration observed at the Day 15 timepoint (Figure 5F), these long‐term outcomes support the notion that the sequential processes underlying the proposed dual‐targeting mechanism, namely NK cell recruitment, M1EV‐mediated bridge‐like NK‐tumor cell association, NK cell activation, and tumor cell apoptosis, progressively contribute to durable tumor growth suppression, and further support an enhanced combined contribution of HA‐ and cRGD‐mediated targeting to the overall antitumor efficacy of the platform.

## 4 Conclusions

In this study, innovative EV‐based biomaterials derived from M1 macrophages were developed to reprogram TME and enhance the efficacy of NK cell‐mediated cancer immunotherapy. Our results confirmed that the isolated M1EVs preserved the pro‐inflammatory characteristics of M1 macrophages. Dual‐engineered M1EV_HA/cRGD, functionalized with HA and cRGD, promoted receptor‐assisted association with NK cells and MCF‐7 breast cancer cells under the tested conditions. Fluorescence‐based assessment further showed that both HA and cRGD were retained in the final formulation after surface modification and purification, supporting successful dual‐ligand engineering of M1EVs.

M1EV_HA/cRGD facilitated cluster formation between NK and MCF‐7 cells, which was accompanied by increased NK effector molecule secretion and a moderate enhancement of early NK‐mediated tumor cell lysis. *In vivo* studies using an orthotopic cancer model demonstrated the antitumor efficacy of both M1EV and M1EV_HA/cRGD, with M1EV_HA/cRGD showing enhanced therapeutic effects compared with non‐engineered M1EV.

Although M1EV_HA/cRGD enhanced association with both NK and tumor cells and increased NK‐tumor cell clustering, the present data do not directly visualize a single EV simultaneously engaging both cell types. The proposed bridge‐like function of M1EV_HA/cRGD should therefore be interpreted as a mechanism supported by differential receptor expression profiling, selective NK‐tumor cell clustering over NK‐HMEC clustering, early AKT pathway activation in NK‐92 MI cells following M1EV_HA/cRGD treatment, and cellular association and clustering assays, rather than as direct single‐vesicle‐level evidence of HA‐CD44/cRGD‐integrin‐mediated bridging. Future studies incorporating free HA/free cRGD competition within NK‐tumor co‐cultures, together with high‐resolution live‐cell imaging or single‐particle tracking, will be required to fully validate this proposed mechanism.

Although the dual‐ligand design enhanced receptor‐assisted NK‐tumor cell association, CD44 and integrins are broadly expressed in various normal tissues. Therefore, systemically administered M1EV_HA/cRGD may also interact with non‐tumor cells to some extent. In the present study, H&E staining of major organs revealed no apparent histopathological abnormalities following M1EV_HA/cRGD administration, suggesting that potential non‐specific interactions did not cause detectable organ toxicity under the tested dosing regimen. Considering this analysis does not directly quantify EV biodistribution or off‐target cell binding, future studies using fluorescently or radiolabeled EV tracking and quantitative organ‐level accumulation analysis will be required to further define the biodistribution and off‐target interaction profile of M1EV_HA/cRGD.

In summary, this study highlights the potential of dual‐targeted M1EVs as a receptor‐assisted and immunomodulatory EV platform for cancer immunotherapy and offers a promising strategy to enhance NK cell‐based therapeutic responses against solid tumors.

## Author Contributions

**Su Jin Kang** and **Gichan Baek** contributed equally to this work and are the first co‐authors. Su Jin Kang: Writing – Original draft, Conceptualization, Methodology, Visualization, Validation, Formal Analysis, Investigation, Writing – Review and editing. Gichan Baek: Methodology, Visualization, Validation, Formal Analysis, Writing – Review and editing. **Suwun Ju**: Formal Analysis. **Gunhee Kim**: Formal Analysis. **Seongjin Gwak**: Formal Analysis. **Won Jong Rhee**: Writing – original draft, Conceptualization, Project administration, Formal Analysis, Resources, Supervision, Funding acquisition.

## Funding

This work was supported by the National Research Foundation of Korea (NRF) Grant funded by the Korean Government (RS‐2022‐NR069930, RS‐2025‐02263404). This work was supported by Korea Polar Research Institute (KOPRI) grant funded by the Ministry of Oceans and Fisheries (KOPRI PE25900).

## Ethics statement

All procedures complied with the standards of the Experimental Ethics Committee (INU‐ANIM‐2024‐0035).

## Conflicts of Interest

The authors declare no conflicts of interest.

## Supporting information




**Supporting Information**: jev270379‐sup‐0001‐SuppMat.docx

## Data Availability

The data that support the findings of this study are available from the corresponding author upon reasonable request.
