# Supplementary material for: Dual‐Targeted M1 Macrophage Extracellular Vesicles Reprogram the Tumor Microenvironment and Enhance Natural Killer Cell Immunotherapy in Breast Cancer
Source: J Extracell Vesicles. 2026 Sep 30;15(10):e70379. doi: 10.1002/jev2.70379 (PMC13626480; doi:10.1002/jev2.70379)
Supplement: Supplementary file 1 [file JEV2-15-e70379-s001.docx]

Supplementary information

**Dual-Targeted M1 Macrophage Extracellular Vesicles Reprogram the Tumor Microenvironment and Enhance Natural Killer Cell Immunotherapy in Breast Cancer**

Su Jin Kang ^1,a^, Gichan Baek ^1,2,a^, Suwun Ju ^1,2^, Gunhee Kim^1,2^, Seongjin Gwak ^1,2^, Won Jong Rhee ^1,2,3,4*^

1. Department of Bioengineering and Nano-Bioengineering, Incheon National University Incheon 22012, Republic of Korea
2. Research Center for Bio Materials & Process Development, Incheon National University, Incheon 22012, Republic of Korea
3. Division of Bioengineering, Incheon National University Incheon 22012, Republic of Korea
4. Integrated Major in Bio-manufacturing AX, Incheon National University, Incheon 22012, Republic of Korea

^a^ Su Jin Kang and Gichan Baek contributed equally to this work and are the first co-authors.

* Corresponding author: Won Jong Rhee, Division of Bioengineering, Incheon National University. Incheon 22012, Republic of Korea. Tel: +82-32-835-8299. Email address: wjrhee@inu.ac.kr (W. J. Rhee)

**Data availability statement:** The data that support the findings of this study are available from the corresponding author upon reasonable request.

**Funding statement:** This work was supported by the National Research Foundation of Korea (NRF) Grant funded by the Korean Government (RS-2022-NR069930, RS-2025-02263404). This work was supported by Korea Polar Research Institute (KOPRI) grant funded by the Ministry of Oceans and Fisheries (KOPRI PE25900).

**Conflict of interest disclosure:** The authors declare no conflicts of interest.

**Ethics approval statement:** All procedures complied with the standards of the Experimental Ethics Committee (INU-ANIM-2024-0035).

**Patient consent statement:** Not applicable.

**Clinical trial registration:** Not applicable.

Supplementary Table and Figure

***Supplementary Table 1***

**Supplementary Table 1.** Primer sequences for qRT-PCR.

| Gene |  | Primer sequence (5’ → 3’) |
| --- | --- | --- |
| Actin | Forward | ATG AAG TGT GAC GTT GAC ATC CG |
|  | Reverse | GCT TGC TGA TCC ACA TCT GCT G |
| CD163 | Forward | GTC GCT CAT CCC GTC AGT CAT C |
|  | Reverse | GCC GCT GTC TCT GTC TT |
| IL-1β | Forward | GCT GAT GGC CCT AAA CAG A |
|  | Reverse | TCG GAG ATT CGT AGC TGG AT |
| IFN-γ | Forward | CTG TTA CTG CCA GGA CCC AT |
|  | Reverse | TCT GTC ACT CTC CTC TTT CCA A |
| Granzyme B | Forward | TGC AGG AAG ATC GAA AGT GCG |
|  | Reverse | GAG GCA TGC CAT TGT TTC GTC |
| Perforin | Forward | CAG TAC AGC TTC AGC ACT GAC |
|  | Reverse | ATG AAG TGG GTG CCG TAG TTG |

***Supplementary Table 2***

**Supplementary Table 2.** Stepwise procedure for THP-1 differentiation, M1-like polarization, and phenotype validation before M1EV isolation.

| Step | Conditions | Purpose | Observation |
| --- | --- | --- | --- |
| Monocyte  maintenance | Suspension culture | Maintenance of THP-1 monocytes  before differentiation | Round,  suspension cells |
| Macrophage-like  differentiation | 200 ng/mL  PMA, 24 h | Induction of adherent  macrophage-like phenotype | Increased  adherent morphology |
| Resting | Fresh complete  medium, 24 h | Reduction of acute PMA-induced activation | Stabilized  adherent morphology |
| M1-like polarization | 1,000 ng/mL LPS +  20 ng/mL IFN-γ, 72 h | Induction of M1-like macrophage phenotype | Significantly increased  CD86 expression;  CD206 remained low |
| Phenotype validation | Flow cytometry  For CD14/CD86/CD206 | Confirmation of monocyte/macrophage  lineage and  polarization status | CD14 lineage marker profile, CD86-high  M1-like profile,  and low CD206 pattern |

***Supplementary Table 3***

**Supplementary Table 3.** Statistical comparison of tumor volume among treatment groups over the extended *in vivo* study period (related to Figure 6D).

| Comparison | D0-D24 | D27 | D30 |
| --- | --- | --- | --- |
| CTRL vs M1EV | n.s | p = 0.01 | p = 0.008 |
| CTRL vs M1EV_HA | n.s | p = 0.02 | p = 0.02 |
| CTRL vs M1EV_cRGD | n.s | p = 0.008 | p = 0.04 |
| CTRL vs M1EV_HA/cRGD | n.s | p < 0.001 | p < 0.001 |
| M1EV vs M1EV_HA | n.s | n.s | n.s |
| M1EV vs M1EV_cRGD | n.s | n.s | n.s |
| M1EV vs M1EV_HA/cRGD | n.s | n.s | n.s |
| M1EV_HA vs M1EV_cRGD | n.s | n.s | n.s |
| M1EV_HA vs M1EV_HA/cRGD | n.s | n.s | n.s |
| M1EV_cRGD vs M1EV_HA/cRGD | n.s | n.s | n.s |

***Figure S1***

**Supplementary Figure 1. Flow cytometric validation of THP-1-derived macrophage differentiation and M1-like polarization prior to M1EV isolation.** THP-1 monocytes, PMA-differentiated M0 macrophage-like cells, and LPS/IFN-γ-polarized M1-like macrophages were analyzed for the expression of (A) CD14, (B) CD86, and (C) CD206. The percentage of CD14-positive cells was significantly increased in the M0 macrophage-like group, consistent with PMA-induced macrophage-like differentiation. The percentage of CD86-positive cells was significantly increased in the M1-like group, whereas CD206 expression remained low across all groups, indicating the absence of M2-like polarization. Data are presented as mean ± SD, with n ≥ 3. Statistical significance: ***p < 0.001; n.s, not significant.

***Figure S2***

**Supplementary Figure 2. Stability of M1EV under different storage conditions.** M1EVs were stored in PBS at 2-8 °C (A) or 37 °C (B) for 7 days. The particle size and concentration of M1EVs were monitored using NTA. Data are presented as mean ± SD, with n ≥ 3. Statistical significance: ***p < 0.001; n.s, not significant.

***Figure S3***

**Supplementary Figure 3. Biocompatibility assessment of M1EV.** Hemolysis was determined by measuring the absorbance of supernatants at 414 nm and 655 nm after centrifugation of red blood cell suspensions. PBS was used for negative CTRL, and nuclease-free water was used for positive CTRL. M1EVs were then added, and the mixture was incubated at 37°C for 1 h. Data are presented as mean ± SD, with n ≥ 3. Statistical significance: ***p < 0.001. The hemolysis rate was calculated as {[(OD_414_ - OD_655_) _sample_ – (OD_414_ - OD_655_) _negative control_] / [(OD_414_ - OD_655_) _positive control_ – (OD_414_ - OD_655_) _negative control_]} × 100.

***Figure S4***

**Supplementary Figure 4. MALDI-TOF mass spectra of cRGD, DSPE-PEG-COOH, and DSPE-PEG-cRGD.** From top to bottom: the free cRGD peptide, showing a dominant peak at m/z 948.402 consistent with its expected molecular weight ([M+H]^+^); DSPE-PEG-COOH; and the purified, lyophilized DSPE-PEG-cRGD conjugate, dissolved in deionized water immediately prior to measurement, showing a systematic shift of the mass envelope toward higher m/z values relative to DSPE-PEG-COOH, corresponding to an apparent mass increase of 926.8 ± 0.7 Da.

***Figure S5***

**Supplementary Figure 5. Critical micelle concentration analysis of DSPE-PEG-HA and DSPE-PEG-cRGD.** Critical micelle concentration **(**CMC) values of the amphiphilic DSPE-PEG-based ligands were determined using pyrene as a hydrophobic fluorescent probe. The pyrene fluorescence intensity ratio of the third to first emission peak (I_3_/I_1_) was plotted as a function of DSPE-PEG-HA or DSPE-PEG-cRGD concentration. The estimated CMC values were 0.037 mg/mL for DSPE-PEG-HA and 0.022 mg/mL for DSPE-PEG-cRGD. Dashed lines indicate the calculated CMC values.

***Figure S6***

**Supplementary Figure 6. Fluorescence-based assessment of HA incorporation onto engineered M1EV_HA/cRGD.** (A) DSPE-PEG-HA was labeled with Alexa Fluor^TM^ 647-Hydrazide through EDC/NHS-mediated conjugation and used to quantify HA incorporation after surface modification. (B) Fluorescence intensity analysis showed that the control and unmodified M1EV groups exhibited low background signals. In contrast, M1EV_HA/cRGD showed a markedly increased HA-associated fluorescence intensity, supporting the incorporation of DSPE-PEG-HA onto engineered M1EVs. Data are presented as mean ± SD, with n ≥ 3. Statistical significance: ***p < 0.001.

***Figure S7***

**Supplementary Figure 7. Enhanced cellular association and internalization of M1EV_HA/cRGD by MCF-7 cells.** Confocal microscopy images showing cellular association/internalization of BODIPY_FL-labeled M1EV, and M1EV_HA/cRGD in MCF-7 cells in comparison with the untreated control (CTRL). Blue, green, and red fluorescence represent nuclei, BODIPY_FL-labeled EVs, and Alexa Fluor^®^ 594 Phalloidin-stained F-actin, respectively; scale bar, 50 μm.

***Figure S8***

**Supplementary Figure 8. Receptor-blocking assay for M1EV_HA/cRGD association with MCF-7 cells.** Confocal microscopy images of fluorescently labeled M1EV and M1EV_HA/cRGD association with MCF-7 cells in the absence (w/o blocking) or presence (w/ blocking) of anti-integrin αvβ5 antibody blocking. MCF-7 cells were pretreated with an anti-integrin αvβ5 antibody prior to incubation with fluorescently labeled EV formulations. M1EV_HA/cRGD exhibited strong association with MCF-7 cells under non-blocking conditions, whereas anti-integrin blocking markedly reduced the EV-derived fluorescence signal, supporting the involvement of cRGD-integrin interaction in tumor cell association. Blue, green, and red fluorescence represent nuclei (Hoechst 33342), BODIPY™ FL-labeled EVs, and Alexa Fluor^®^ 594 Phalloidin-stained F-actin, respectively; scale bars, 50 μm.

***Figure S9***

**Supplementary Figure 9. Characterization of CD44 and integrin αvβ5 expression in NK-92 MI cells and breast cancer-related cell lines.** (A) Flow cytometric analysis of CD44 expression in HMEC and NK-92 MI cells. Representative histograms show markedly higher CD44-associated fluorescence in NK-92 MI cells compared with HMECs. (B) Western blot analysis of CD44 expression in HMECs and NK-92 MI cells, confirming the flow cytometry results. GAPDH was used as a loading control. (C) Flow cytometric analysis of integrin αvβ5 expression in HMECs and MCF-7 cells. Representative histograms and quantitative analysis show significantly higher integrin αvβ5 expression in MCF-7 cells compared with HMECs. Data are presented as mean ± SD with n ≥ 3. Statistical significance: ***p < 0.001.

***Figure S10***

**Supplementary Figure 10. AKT phosphorylation in NK-92 MI cells following M1EV_HA/cRGD treatment.** (A) Western blot analysis of AKT and phospho-AKT (p-AKT) expression in NK-92 MI cells co-cultured with MCF-7 cells at an E:T ratio of 1:2 in the absence (CTRL) or presence of M1EV or M1EV_HA/cRGD (5 × 10^9^ particles/mL) for 15 min. NC denotes NK-92 MI cells cultured alone without MCF-7 cells or EV treatment. (B) Quantification of p-AKT/AKT ratio by densitometric analysis, normalized to the CTRL group. Data are presented as mean ± SD, with n ≥ 3. Statistical significance: *p < 0.05; **p < 0.01.

***Figure S11***

**Supplementary Figure 11. Quantitative analysis of Ki-67 and CD56 expression in tumor tissues at Day 15.** (A) Quantification of Ki-67-positive area (%) in tumor tissue sections from CTRL, M1EV, and M1EV_HA/cRGD-treated groups based on IHC-stained images (Figure 5E). (B) Quantification of CD56-positive area (%) in tumor tissue sections from CTRL, M1EV, and M1EV_HA/cRGD-treated groups based on IF-stained images (Figure 5F). Data are presented as mean ± SD, with n ≥ 3. Statistical significance: *p < 0.05; **p < 0.01; ***p < 0.001.

***Figure S12***

**Supplementary Figure 12. Quantitative analysis of Ki-67 and TUNEL staining in tumor tissues at Day 30.** (A) Quantification of Ki-67-positive area (%) in tumor tissue sections from CTRL, M1EV, M1EV_HA, M1EV_cRGD, and M1EV_HA/cRGD-treated groups based on IHC-stained images (Figure 6F). (B) Quantification of TUNEL-positive area (%) in tumor tissue sections from the same groups based on TUNEL-stained images (Figure 6G). Data are presented as mean ± SD, with n ≥ 3. Statistical significance: *p < 0.05; **p < 0.01; ***p < 0.001; n.s., not significant.
